# Clinical Spectrum and Organ Involvement in Pediatric Melioidosis: A Systematic Review and Meta-Analysis

**DOI:** 10.3390/life16091555

**Published:** 2026-09-16

**Authors:** Jongkonnee Thanasai, Anchalee Chittamma, Supphachoke Khemla, Atthaphong Phongphithakchai, Moragot Chatatikun, Jitbanjong Tangpong, Sa-ngob Laklaeng, Jirarat Songsri, Wiyada Kwanhian Klangbud

**Affiliations:** 1 Faculty of Medicine, Mahasarakham University, Mahasarakham 44000, Thailand; jongkonnee@msu.ac.th; 2 Department of Pathology, Faculty of Medicine Ramathibodi Hospital, Mahidol University, Bangkok 10400, Thailand; anchalee.chi@mahidol.ac.th; 3 Division of Infectious Diseases, Department of Internal Medicine, Nakhon Phanom Hospital, Nakhon Phanom 48000, Thailand; sup.mednkp@gmail.com; 4 Nephrology Unit, Division of Internal Medicine, Faculty of Medicine, Prince of Songkla University, Songkhla 90110, Thailand; atthaphong.p@psu.ac.th; 5 School of Allied Health Sciences, Walailak University, Nakhon Si Thammarat 80160, Thailand; moragot.ch@wu.ac.th (M.C.); rjitbanj@wu.ac.th (J.T.); sangob.ll@wu.ac.th (S.-n.L.); jirarat.so@wu.ac.th (J.S.); 6 Medical Technology Program, Faculty of Science, Nakhon Phanom University, Nakhon Phanom 48000, Thailand; 7 Faculty of Medicine, Nakhon Phanom University, Nakhon Phanom 48000, Thailand

**Keywords:** *Burkholderia pseudomallei*, melioidosis, pediatric infectious diseases, clinical manifestations, organ involvement, bacteremia, mortality

## Abstract

**Background:** Pediatric melioidosis causes substantial morbidity and mortality in tropical regions, yet its clinical spectrum has not been comprehensively quantified. We conducted a systematic review and meta-analysis to characterize clinical manifestations, organ involvement, and mortality among children with melioidosis. **Methods:** PubMed, Embase, and Scopus were searched from inception to 31 May 2026 for observational studies reporting clinical manifestations or outcomes in children (<18 years) with melioidosis. Pooled prevalence estimates and case-fatality rates were calculated using random-effects generalized linear mixed models with logit transformation. Heterogeneity was assessed using Cochran’s Q, *I*^2^, and τ^2^. Country subgroup analyses were performed, and small-study effects were assessed for outcomes with at least ten studies. **Results:** Twenty-five studies involving 6874 children from 10 countries were included. Localized infection predominated (69%; 95% CI, 52–82%), whereas disseminated infection occurred in 30% (95% CI, 18–46%). Skin and soft tissue infection was the most frequent manifestation (31%; 95% CI, 23–41%), followed by lymphadenitis (24%; 95% CI, 3–76%), pneumonia (18%; 95% CI, 17–19%), and parotitis (13%; 95% CI, 5–28%). Bacteremia occurred in 23% (95% CI, 11–43%), septic shock in 6% (95% CI, 0–53%), and the pooled case-fatality rate was 12% (95% CI, 7–20%). Significant geographic variation was observed for localized infection, disseminated infection, pneumonia, bacteremia, and mortality (all *p* < 0.01). Small-study effects were detected for several outcomes. **Conclusions:** Pediatric melioidosis predominantly presents as localized disease, but disseminated infection, bacteremia, and mortality remain clinically important. Geographic variation and substantial heterogeneity highlight differences in disease presentation across settings. These findings provide quantitative evidence to support earlier recognition, diagnostic evaluation, and timely management of pediatric melioidosis in endemic regions.

## 1. Introduction

Melioidosis is a potentially fatal infectious disease caused by the environmental Gram-negative bacterium *Burkholderia pseudomallei*, which is widely distributed in soil and surface water throughout tropical and subtropical regions. The disease is highly endemic in Southeast Asia and northern Australia, with increasing recognition in South Asia, Africa, and the Americas as surveillance and diagnostic capacity improve [1,2]. Recent modelling studies estimate that melioidosis causes approximately 165,000 human cases and 89,000 deaths annually, making it one of the leading causes of death from bacterial infections in tropical regions [3]. Despite this considerable disease burden, melioidosis remains substantially underdiagnosed and underreported because of limited microbiological capacity, nonspecific clinical manifestations, and low disease awareness in many endemic settings [1,2].

Although melioidosis has traditionally been regarded as a disease affecting adults with underlying comorbidities, pediatric melioidosis is increasingly recognized as a distinct clinical entity with unique epidemiological and clinical characteristics [2,4]. Children commonly acquire infection through direct contact with contaminated soil or surface water, inhalation, or ingestion, particularly during the rainy season. Compared with adults, pediatric patients are more likely to present with localized disease, especially suppurative parotitis, skin and soft tissue infection, and cervical lymphadenitis, whereas pneumonia, septic shock, and extensive multi-organ involvement are more frequently observed in adults [1,4]. Nevertheless, severe disseminated disease with bacteremia, visceral abscesses, neurological involvement, osteoarticular infection, and death may also occur in children, particularly when diagnosis is delayed or in those with underlying medical conditions [2].

The clinical spectrum of pediatric melioidosis is highly heterogeneous, ranging from uncomplicated cutaneous infection to life-threatening septicemia involving multiple organ systems, including the lungs, liver, spleen, bones, joints, central nervous system, and genitourinary tract [1,2]. However, the reported frequency of these manifestations varies considerably across geographic regions and study populations owing to differences in environmental exposure, healthcare access, diagnostic practices, and study design. This variability complicates clinical recognition and may contribute to delayed diagnosis, particularly in non-endemic areas where clinician awareness remains limited.

Several observational studies and case series have described the clinical characteristics of pediatric melioidosis; however, the available evidence remains fragmented. McLeod et al. (2015) characterized childhood melioidosis in northern Australia and demonstrated that localized infections predominate, although severe disseminated disease remains an important cause of morbidity and mortality [4]. Wiersinga et al. (2012) comprehensively reviewed the epidemiology, pathogenesis, diagnosis, and management of melioidosis but focused primarily on the disease across all age groups rather than on the pediatric population [1]. More recently, Meumann et al. (2024) summarized current advances in melioidosis research and highlighted the distinct features of pediatric infection [2]. A recent pediatric-specific scoping review by Vithiya et al. (2026) synthesized 140 reports comprising 1058 pediatric cases and described the broad phenotype-severity continuum of pediatric melioidosis, including the contrasting outcomes of localized, disseminated, neonatal, and neurological disease [5]. However, that review used a narrative scoping approach and included case reports and case series; it did not provide random-effects pooled prevalence estimates for individual clinical manifestations and organ involvement. Consequently, important uncertainties remain regarding the pooled frequency and between-study variation of major pediatric manifestations and outcomes.

A comprehensive quantitative synthesis of the available evidence is therefore needed to improve clinical recognition, inform diagnostic evaluation, and support timely management of pediatric melioidosis. Reliable estimates of the prevalence of specific clinical manifestations may also guide decisions regarding microbiological investigations, imaging strategies, and clinical monitoring, particularly in endemic regions where delayed diagnosis is associated with increased morbidity and mortality.

Therefore, the present systematic review and meta-analysis aims to comprehensively characterize the clinical spectrum of pediatric melioidosis by estimating the pooled prevalence of major clinical manifestations, organ involvement, and important clinical outcomes among children and adolescents with melioidosis. Specifically, this study evaluates the prevalence of parotitis, skin and soft tissue infection, pneumonia, lymphadenitis, osteomyelitis, septic arthritis, neurological involvement, hepatic involvement, splenic involvement, bacteremia, disseminated infection, and mortality. By quantitatively integrating evidence from diverse geographic settings and study populations, this review seeks to provide robust evidence to facilitate earlier diagnosis, improve clinical management, and identify priorities for future research in pediatric melioidosis.

## 2. Materials and Methods

### 2.1. Study Design and Protocol Registration

This systematic review and meta-analysis followed the Preferred Reporting Items for Systematic Reviews and Meta-Analyses (PRISMA) 2020 Statement [6]. The review protocol was prospectively registered with the International Prospective Register of Systematic Reviews (PROSPERO registration no. CRD420261411428) before study selection commenced; the review aimed to characterize the clinical spectrum, organ involvement, and outcomes of pediatric melioidosis. During the conduct of the review, the analytic approach was refined to include quantitative random-effects meta-analysis where sufficiently comparable data were available, all-cause mortality as a secondary outcome, and assessment of small-study effects for outcomes reported by at least 10 studies. These amendments did not alter the review population, eligibility criteria, or core research question and were subsequently documented in the PROSPERO record.

### 2.2. Eligibility Criteria

Studies were eligible if they included participants from birth to <18 years of age, including neonates, newborns, infants, children, and adolescents, with melioidosis attributed to *Burkholderia pseudomallei*. Culture isolation of *B. pseudomallei* from a clinical specimen was considered the reference standard for diagnosis [1,2]. Molecular confirmation of *B. pseudomallei* was also accepted for use on culture isolates or clinical specimens. Because this review spans several decades and includes surveillance studies conducted under historical diagnostic frameworks, we did not impose a single retrospective case definition across all studies; instead, the diagnostic criteria reported in each original study were explicitly recorded. Studies using mixed diagnostic approaches (e.g., culture and serology) or administrative coding were retained when they met the prespecified eligibility criteria, but their diagnostic uncertainty was considered in the interpretation of findings. Eligible studies were required to report extractable pediatric data on at least one clinical manifestation, organ involvement, bacteremia, disseminated infection, mortality, or other clinical outcomes. Observational study designs, including prospective and retrospective cohort studies, cross-sectional studies, and surveillance studies, were eligible. Studies enrolling exclusively adults, lacking extractable pediatric data, involving animal or laboratory experiments, or consisting of reviews, editorials, commentaries, conference abstracts without sufficient primary data, or duplicate publications were excluded.

### 2.3. Information Sources and Search Strategy

A comprehensive literature search was conducted in PubMed, Embase, and Scopus from database inception through 31 May 2026 without restrictions on language or publication date. The search strategy combined controlled vocabulary and free-text terms related to melioidosis and pediatric populations, including “*Burkholderia pseudomallei*”, “melioidosis”, “child”, “children”, “infant”, “adolescent”, “pediatric”, and related synonyms. Search strategies were adapted appropriately for each database. To maximize study identification, the reference lists of all eligible articles were manually screened, and forward citation tracking was performed to identify additional relevant studies. The details are provided in Supplementary File S1.

### 2.4. Study Selection Procedures

All retrieved records were imported into a reference management program, where duplicate citations were removed before screening. Two reviewers independently screened titles and abstracts according to the predefined eligibility criteria. Potentially relevant articles subsequently underwent full-text assessment by the same reviewers. Any discrepancies regarding study eligibility were resolved through discussion until consensus was reached. The overall selection process was documented using a PRISMA 2020 flow diagram.

### 2.5. Data Extraction

Data were extracted independently by two reviewers using a standardized data extraction form. The extracted information included study characteristics (first author, publication year, country, study design, study period, and sample size), patient demographics (age and sex), underlying medical conditions, clinical manifestations, organ involvement, localized or disseminated infection, bacteremia, diagnostic methods, antimicrobial treatment, and clinical outcomes, including mortality. When studies reported only pediatric subgroups within mixed adult and pediatric populations, only pediatric data were extracted. In accordance with the registered protocol, study authors were not contacted for additional information when published data were incomplete or unavailable.

### 2.6. Quality Assessment

The methodological quality of the included studies was independently assessed by two reviewers using the Newcastle–Ottawa Scale (NOS) [7], with item-level judgments reported in Supplementary Table S1. Because the included evidence consisted predominantly of descriptive single-cohort or surveillance studies without an external comparison group, the NOS items for selection of a non-exposed cohort, comparability of cohorts, and absence of the outcome at baseline were considered structurally not applicable when the study design did not contain the corresponding comparator or longitudinal exposure–outcome framework. An “N/A” designation therefore indicates non-applicability and was not interpreted as a favorable quality judgment or awarded a star. For applicable items, representativeness, diagnostic ascertainment, outcome ascertainment, and follow-up were evaluated from the information reported in each study. Outcome ascertainment was credited when clinical outcomes were obtained from medical records, prospective surveillance, or other clearly described sources. Follow-up duration and completeness were credited only when longitudinal follow-up was relevant and explicitly documented; for in-hospital mortality, follow-up to discharge or death was considered adequate when clearly reported. To provide an overall study-level judgment without treating non-applicable items as favorable, an explicit rule was defined before the present reclassification and applied consistently to all studies: low risk indicated no major concern and no more than one unclear applicable item; moderate risk indicated two or more unclear/minor concerns without a major concern; and high risk indicated at least one major concern, such as a clearly selected/non-representative cohort, substantial diagnostic misclassification risk from non-microbiological case definitions, or multiple serious limitations. Disagreements were resolved by discussion until consensus was reached.

### 2.7. Outcomes

The primary outcomes were the prevalence of clinical manifestations and organ involvement among children and adolescents with melioidosis. Clinical manifestations of interest included parotitis, skin and soft tissue infection, pneumonia, lymphadenitis, osteomyelitis, septic arthritis, neurological involvement, hepatic involvement, splenic involvement, bacteremia, and disseminated infection. The secondary outcome was all-cause mortality, expressed as the case-fatality rate.

### 2.8. Statistical Analysis

Statistical analyses were performed using R software (version 4.5.0; R Foundation for Statistical Computing, Vienna, Austria) with the meta package. Pooled prevalence estimates and corresponding 95% confidence intervals (CIs) were calculated using random-effects generalized linear mixed models (GLMMs) with a logit transformation (PLOGIT) to stabilize the variance of prevalence estimates. Between-study variance (τ^2^) was estimated using the maximum likelihood (ML) method, and 95% CIs for pooled estimates were calculated using the Hartung–Knapp adjustment. Statistical heterogeneity was assessed using Cochran’s Q test, the *I*^2^ statistic, and τ^2^, with *I*^2^ values >50% indicating substantial heterogeneity. Prespecified subgroup analyses according to country were performed for outcomes with sufficient data to explore potential geographic variation. For outcomes including at least 10 studies, small-study effects were assessed by visual inspection of funnel plots and Egger’s linear regression test for funnel plot asymmetry. All statistical tests were two-sided, and a *p* value < 0.05 was considered statistically significant.

## 3. Results

### 3.1. Study Selection

The literature search identified 1640 records from three electronic databases, including 455 records from PubMed, 646 from Scopus, and 539 from Embase. After removing 630 duplicate records, 1010 unique records remained for title and abstract screening. During this stage, 789 records were excluded because they were conference abstracts *(n* = 10), reviews or systematic reviews (*n* = 18), experimental studies (*n* = 13), animal studies (*n* = 10), or were not relevant to the review question (*n* = 738).

**Figure 1:**
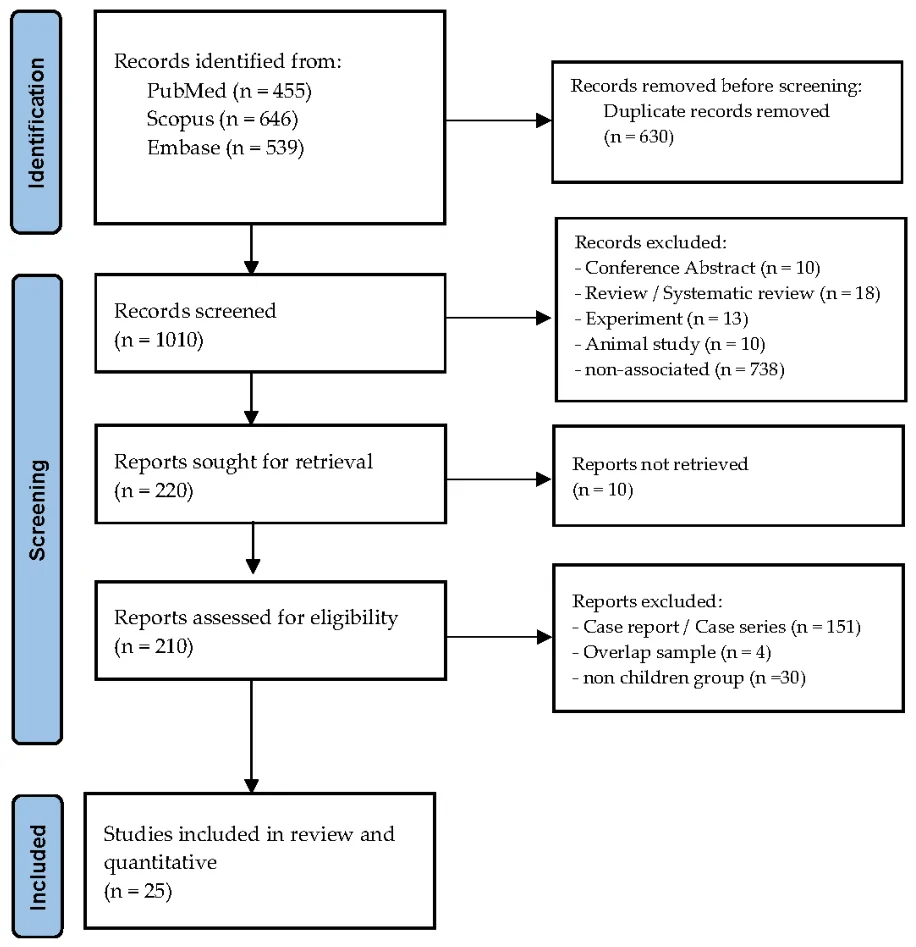
PRISMA flow.

A total of 220 full-text articles were sought for retrieval, of which 10 reports could not be retrieved. Consequently, 210 full-text articles were assessed for eligibility. Of these, 185 studies were excluded because they were case reports or case series (*n* = 151), contained overlapping study populations (*n* = 4), or did not provide data specific to pediatric patients (*n* = 30). Ultimately, 25 studies met the eligibility criteria and were included in the systematic review and quantitative synthesis (Figure 1).

### 3.2. Characteristics of Included Studies

The characteristics of the 25 included studies are summarized in Table 1. The studies were published between 1988 and 2026 and were conducted across 10 countries, including Thailand (*n* = 7), Cambodia (*n* = 3), Malaysia *(n* = 3), Australia (*n* = 3), India (*n* = 2), Singapore (*n* = 2), and one study each from Brazil, Indonesia, Laos, and Vietnam. Most studies employed retrospective cohort or surveillance designs, although several prospective cohorts and one ambispective interventional study were also included. Together, the studies comprised 6874 pediatric patients with confirmed or clinically diagnosed melioidosis, with individual study sample sizes ranging from 5 to 5044 patients.

The reported age of participants varied considerably across studies. Most studies included children younger than 15 or 18 years, with median ages generally ranging from 5 to 8 years, indicating that pediatric melioidosis predominantly affects young children. Several studies reported age as ranges or age categories rather than summary statistics. Male predominance was observed in studies reporting sex distribution, with the proportion of male patients ranging from approximately 45% to 86%. Diagnosis was primarily established by culture confirmation, although several studies also incorporated serological testing, molecular confirmation, or accepted microbiological diagnostic criteria. One nationwide administrative study identified patients using ICD (International Classification of Diseases)-coded diagnoses. After rechecking the diagnostic methods, 20 of the 25 included studies were based entirely on culture-confirmed or otherwise microbiologically confirmed *B. pseudomallei* infection. Five studies used mixed or nonuniform case definitions, including combinations of culture and serology, historical surveillance definitions, or administrative ICD coding. These diagnostic approaches are specified in Table 1 to distinguish microbiologically confirmed cohorts from studies with greater diagnostic uncertainty.

### 3.3. Clinical Manifestations and Outcomes

The clinical manifestations reported by the included studies are summarized in Table 2. Considerable heterogeneity was observed in disease presentation across studies. Localized melioidosis was generally more common than disseminated disease, although the reported proportions varied substantially according to study population and disease severity. Several hospital-based cohorts reported that 60–100% of children presented with localized infection, whereas studies focusing on severe hospitalized cases reported substantially higher frequencies of disseminated disease.

Among localized manifestations, parotitis, skin and soft tissue infection (SSTI), and lymphadenitis were the most frequently reported presentations. The prevalence of parotitis ranged from 14.8% to 42.9% among studies reporting this outcome, while skin and soft tissue infections occurred in 5.0% to 60.0% of pediatric patients. Lymphadenitis was reported in up to 95.0% of children in one Malaysian cohort but was less frequently described in most other studies. Pneumonia was one of the most common systemic manifestations, with reported frequencies ranging from 5.0% to 90.0%, particularly among cohorts enriched for severe disease. Neurological involvement was less common, generally affecting 2.6–20.0% of patients. Hepatic and splenic involvement were reported inconsistently across studies but reached frequencies of approximately 30% in some cohorts. Bacteremia, when reported, ranged from 3.6% to 100%, reflecting differences in study populations and disease severity. Septic shock was documented in up to 80.0% of patients in severe hospital-based cohorts but remained uncommon in population-based studies.

### 3.4. Risk-of-Bias Assessment

The item-level and overall risk-of-bias assessments are presented in Supplementary Table S1. The “N/A” ratings for the non-exposed cohort, baseline outcome, and comparability items reflect structural non-applicability to descriptive single-cohort designs and should not be interpreted as favorable assessments. Using the explicit study-level decision rule based only on applicable domains, 3 studies were judged to be at low overall risk of bias, 14 at moderate risk, and 8 at high risk.

Studies judged to be at high risk were primarily those with selected clinical or imaging-based cohorts that limited representativeness, or those using mixed serological, surveillance, or administrative case definitions with greater potential for diagnostic misclassification. Most moderate-risk studies used microbiological confirmation and adequate outcome ascertainment but had incomplete reporting of representativeness or follow-up. The low-risk studies had representative or well-defined cohorts, microbiological confirmation, clear outcome ascertainment, and adequate follow-up information. These judgments were used to inform interpretation rather than to exclude studies solely on the basis of quality.

### 3.5. Meta-Analysis of Clinical Manifestations and Organ Involvement

**Figure 2:**
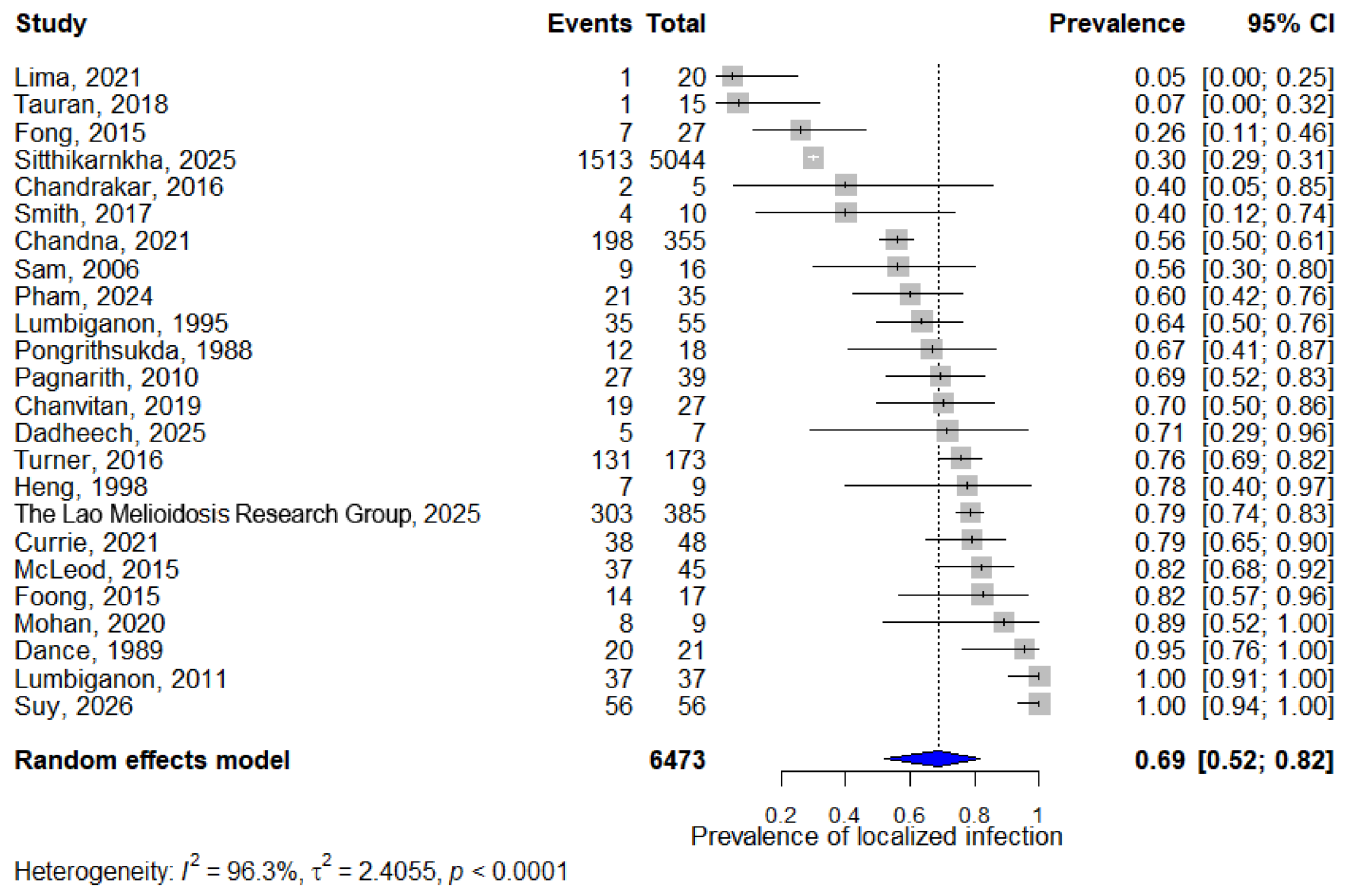
Pooled prevalence of localized infection among children with melioidosis. Study-specific [4,8,9,10,11,12,13,14,15,16,17,18,19,20,22,23,24,25,26,27,28,29,30,31] estimates are displayed by row; the horizontal axis shows prevalence. Gray squares represent study estimates, horizontal lines represent 95% confidence intervals (CIs), and the blue diamond represents the pooled estimate.

**Figure 3:**
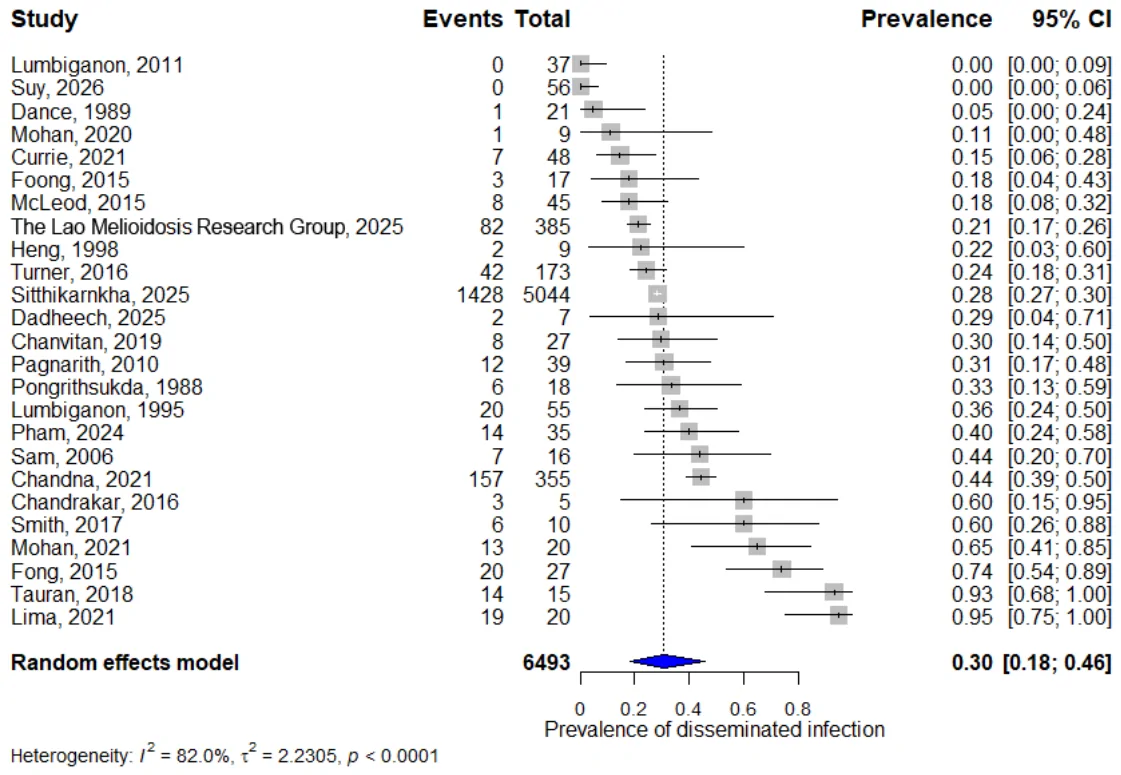
Pooled prevalence of disseminated infection among children with melioidosis. Study-specific [4,8,9,10,11,12,13,14,15,16,17,18,19,20,21,22,23,24,25,26,27,28,29,30,31] estimates are displayed by row; the horizontal axis shows prevalence. Gray squares represent study estimates, horizontal lines represent 95% confidence intervals (CIs), and the blue diamond represents the pooled estimate.

Random-effects meta-analysis demonstrated that localized infection was the predominant clinical presentation in pediatric melioidosis, with a pooled prevalence of 69% (95% CI, 52–82%), whereas disseminated infection occurred in approximately one-third of patients (30%; 95% CI, 18–46%) (Figure 2 and Figure 3). Considerable between-study heterogeneity was observed for both localized (*I*^2^ = 96.3%) and disseminated infection (*I*^2^ = 82.0%).

Among localized manifestations (Supplementary Figures S1.1–S1.9), skin and soft tissue infection was the most frequent presentation, with a pooled prevalence of 31% (95% CI, 23–41%), followed by lymphadenitis (24%; 95% CI, 3–76%), pneumonia (18%; 95% CI, 17–19%), and parotitis (13%; 95% CI, 5–28%). Osteomyelitis and septic arthritis were uncommon, with pooled prevalences of 1% (95% CI, 0–10%) and 1% (95% CI, 0–5%), respectively. Neurological involvement was also infrequent (7%; 95% CI, 3–15%). Hepatic and splenic involvement were reported less consistently across studies, with pooled prevalences of 19% (95% CI, 4–56%) and 86% (95% CI, 0–100%), respectively; however, these estimates were based on only a few studies and should therefore be interpreted cautiously.

**Figure 4:**
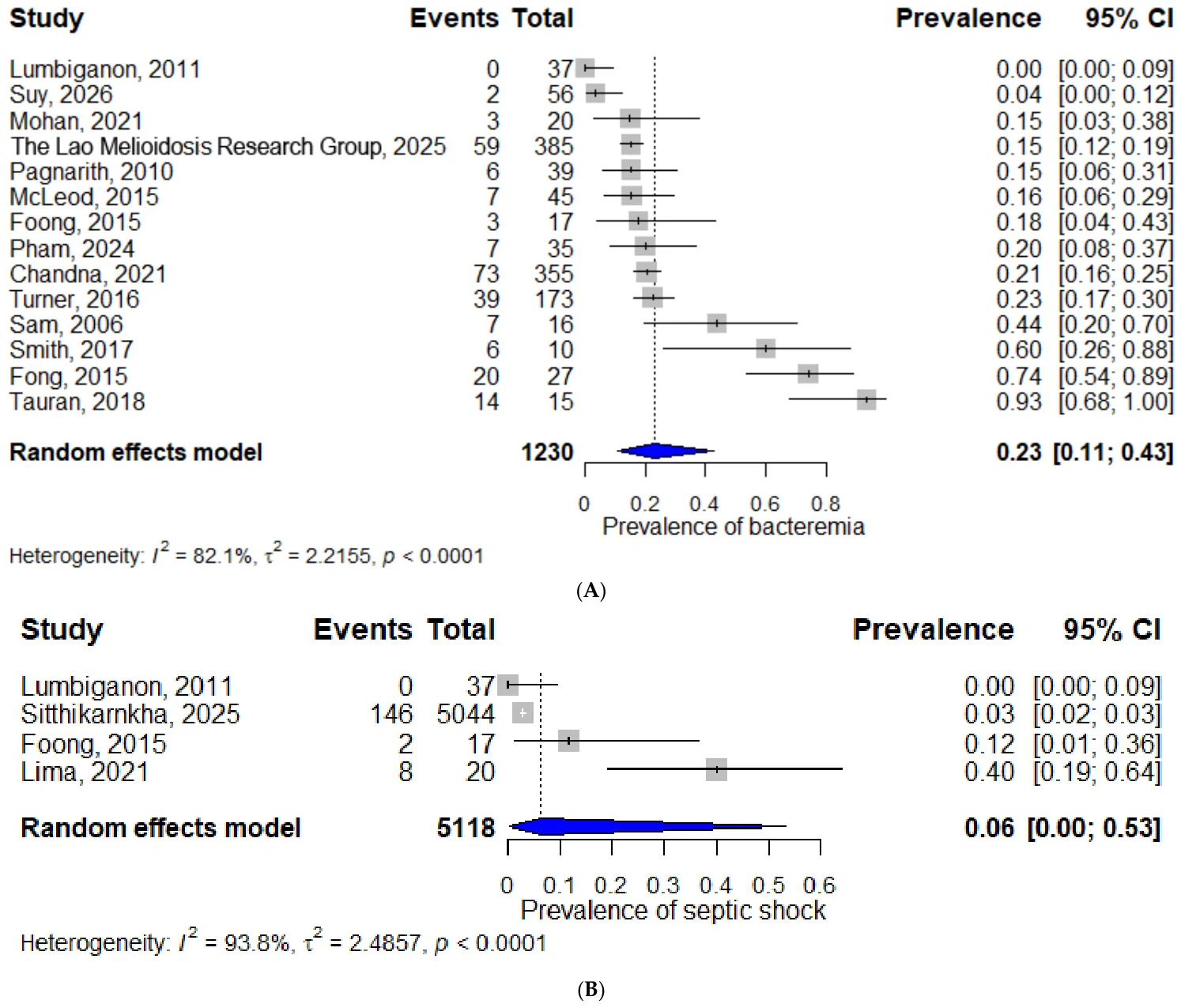
Pooled prevalence of severe clinical manifestations in children with melioidosis: (**A**) bacteremia [4,8,14,15,19,21,22,23,25,26,28,29,30,31] and (**B**) septic shock [15,17,19,27]. Study-specific estimates are displayed by row; the horizontal axis shows prevalence. Gray squares represent study estimates, horizontal lines represent 95% confidence intervals (CIs), and the blue diamond represents the pooled estimate.

**Figure 5:**
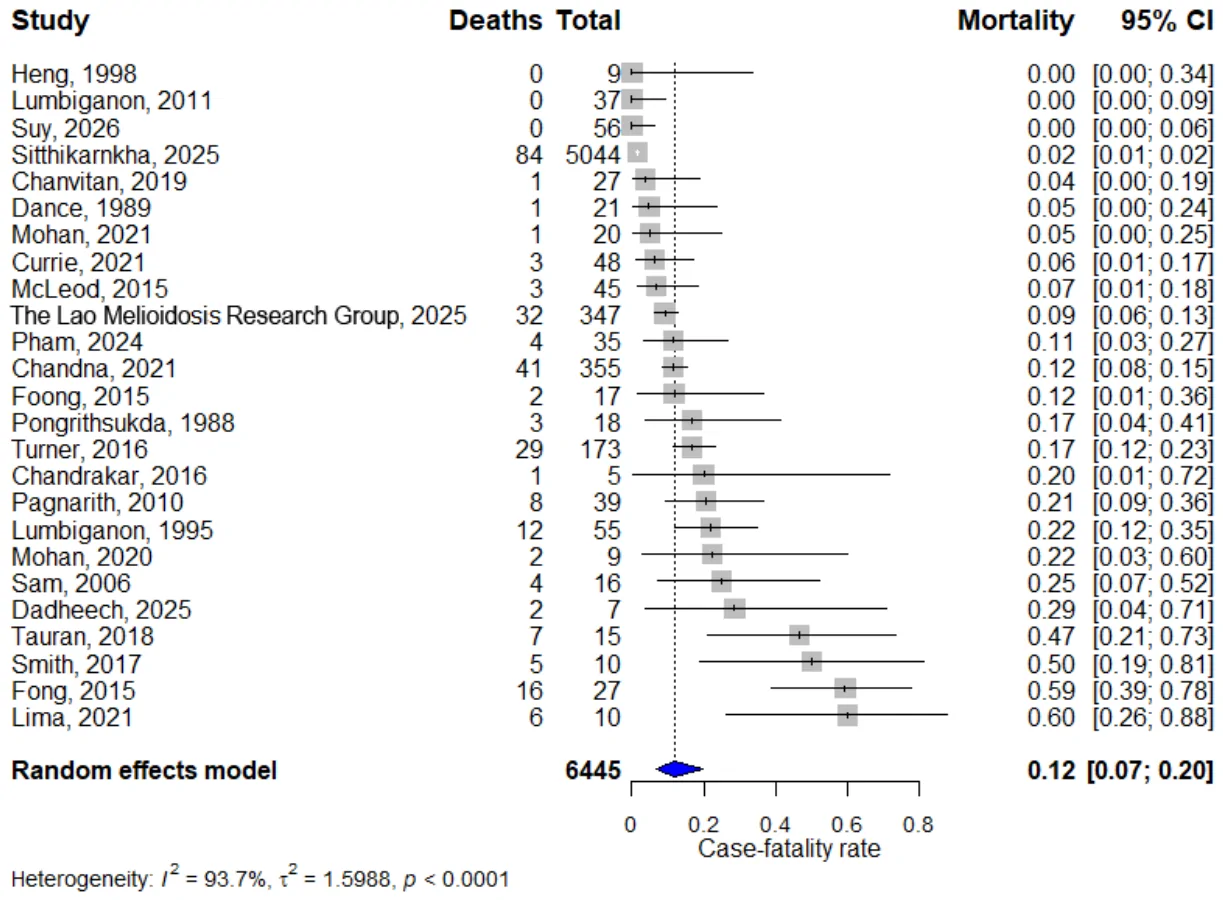
Pooled case-fatality rate among children with melioidosis. Study-specific [4,8,9,10,11,12,13,14,15,16,17,18,19,20,21,22,23,24,25,26,27,28,29,30,31] estimates are displayed by row; the horizontal axis shows prevalence. Gray squares represent study estimates, horizontal lines represent 95% confidence intervals (CIs), and the blue diamond represents the pooled estimate.

Bacteremia was identified in approximately one-quarter of pediatric patients (23%; 95% CI, 11–43%), Figure 4A. Septic shock occurred in 6% (95% CI, 0–53%), Figure 4B. The pooled case-fatality rate was 12% (95% CI, 7–20%), although substantial heterogeneity was observed across studies *(I*^2^ = 93.7%), as shown in Figure 5.

Country subgroup analyses demonstrated significant geographic variation in several major clinical outcomes. The prevalence of localized infection (*p* < 0.0001), disseminated infection (*p* < 0.0001), bacteremia (*p* = 0.0020), pneumonia (*p* = 0.0062), and mortality (*p* < 0.0001) differed significantly among countries (Supplementary Figures S2.1–S2.5). To make these geographic differences more transparent, Table 3 summarizes the study-level ranges of selected pediatric presentations by country. These ranges are descriptive and should not be interpreted as country-level incidence estimates because the contributing studies differed in design, referral setting, diagnostic criteria, and sample size.

### 3.6. Publication Bias

Publication bias was evaluated for outcomes, including at least 10 studies using funnel plots and linear regression tests for funnel plot asymmetry (Supplementary Figures S3.1–S3.13).

Visual inspection of the funnel plots suggested mild asymmetry for localized infection, parotitis, pneumonia, and mortality, whereas disseminated infection, skin and soft tissue infection, and bacteremia appeared relatively symmetrical. Consistent with these observations, the linear regression test demonstrated statistically significant funnel plot asymmetry for localized infection (*p* = 0.0018), parotitis (*p* = 0.0118), pneumonia (*p* = 0.0497), and mortality (*p* = 0.0213), indicating possible small-study effects or publication bias. In contrast, no evidence of funnel plot asymmetry was observed for disseminated infection (*p* = 0.4109), skin and soft tissue infection (*p* = 0.8165), or bacteremia (*p* = 0.3875).

Publication bias was not assessed for lymphadenitis, osteomyelitis, septic arthritis, hepatic involvement, splenic involvement, or septic shock because fewer than 10 studies were available for these outcomes, in accordance with current methodological recommendations.

## 4. Discussion

This systematic review and meta-analysis provides the most comprehensive quantitative synthesis of the clinical spectrum of pediatric melioidosis to date by integrating evidence from 25 studies involving 6874 children across 10 countries. The findings demonstrate that localized infection predominates, affecting approximately two-thirds of pediatric patients, whereas disseminated disease occurs in nearly one-third. Skin and soft tissue infection, lymphadenitis, parotitis, and pneumonia were the most frequently reported clinical manifestations, while neurological disease, osteomyelitis, and septic arthritis were relatively uncommon. Nevertheless, almost one-quarter of children developed bacteremia, and the pooled case-fatality rate remained 12%, highlighting that pediatric melioidosis continues to cause substantial morbidity and mortality despite its predominantly localized presentation.

The predominance of localized disease observed in this review supports previous evidence that pediatric melioidosis differs substantially from adult disease. Adult melioidosis is commonly associated with pneumonia, septicemia, multiorgan abscesses, and septic shock, particularly among patients with diabetes mellitus, chronic kidney disease, excessive alcohol consumption, and other immunocompromising conditions [1,2,11]. In contrast, children generally have fewer chronic comorbidities and are more likely to acquire infection following direct exposure to contaminated soil or surface water through skin inoculation, inhalation, or ingestion during outdoor activities [4]. These differences in host characteristics and exposure pathways may explain the predominance of localized cutaneous and head-and-neck infections in pediatric populations.

The pooled prevalence of localized infection is consistent with observational studies from Thailand, Cambodia, Malaysia, and northern Australia, which have consistently identified skin infection, suppurative parotitis, and lymphadenitis as the most common presentations in children [4,8,18,31]. However, the finding that nearly one-third of children developed disseminated infection indicates that pediatric melioidosis should not be considered a benign disease. Instead, it represents a clinical continuum ranging from uncomplicated localized infection to severe bacteremia with multiple organ involvement. Consequently, children presenting with apparently localized disease should undergo careful clinical assessment for systemic dissemination, particularly when fever persists, inflammatory markers remain elevated, or clinical deterioration occurs despite appropriate empirical antimicrobial therapy.

Skin and soft tissue infection was the most common manifestation identified in the present review. This finding agrees with previous pediatric studies describing cutaneous abscesses, cellulitis, ulcers, and recurrent soft tissue infections as frequent clinical presentations [4,31]. Because these lesions closely resemble infections caused by *Staphylococcus aureus* or *Streptococcus pyogenes*, melioidosis may not initially be suspected, resulting in delayed microbiological investigation and inappropriate empirical treatment. Recent work from Cambodia further demonstrated that rapid microbiological diagnosis of skin and soft tissue melioidosis may facilitate earlier initiation of appropriate therapy [29]. Therefore, melioidosis should be included in the differential diagnosis of recurrent, persistent, or treatment-refractory skin and soft tissue infections in children living in endemic areas.

Suppurative parotitis and cervical lymphadenitis were also common manifestations. Since the first description of pediatric melioidotic parotitis in Thailand, suppurative parotitis has remained one of the hallmark clinical features of childhood melioidosis in Southeast Asia [13]. Subsequent studies from Thailand and Cambodia have consistently reported parotitis as an important localized presentation [18,31]. The pooled prevalence observed in this review confirms its continued diagnostic significance. Likewise, chronic cervical lymphadenitis or recurrent neck abscesses may mimic bacterial adenitis, tuberculosis, or malignancy. Fine-needle aspiration of head-and-neck lesions has been shown to improve microbiological diagnosis and should be considered whenever clinically appropriate [21]. Greater awareness of these characteristic manifestations among frontline clinicians may facilitate earlier diagnosis and reduce progression to invasive disease. A notable geographic contrast is seen in Australia, where acute suppurative parotitis is extremely uncommon and was absent in the pediatric cohorts contributing parotitis data in this review. This contrasts with mainland Southeast Asia, particularly Thailand and Cambodia, where parotitis is a characteristic childhood presentation. Differences in exposure route, including ingestion of contaminated water, may contribute to this pattern; however, bacterial population structure and geographically distributed virulence determinants may also play a role [1,2,32,33].

Although pneumonia affected fewer than one-fifth of children, pulmonary involvement remains one of the most clinically significant manifestations because it is frequently associated with bacteremia, intensive care admission, and mortality [1]. Compared with adults, in whom pneumonia is the dominant presentation, pulmonary disease appears to be proportionally less common in children [2]. Nevertheless, substantial heterogeneity was observed across studies. Hospital-based referral cohorts generally reported higher frequencies of pneumonia than community or population-based studies, suggesting that referral bias, disease severity, imaging availability, and healthcare access substantially influence the reported prevalence. Accordingly, clinicians should continue to consider melioidosis in children with severe community-acquired pneumonia residing in endemic regions, particularly when clinical response to standard empirical therapy is poor.

Approximately 23% of children in this review had bacteremia, indicating that invasive bloodstream infection remains an important determinant of disease severity. Previous studies have consistently demonstrated that bacteremia is associated with disseminated infection, septic shock, and increased mortality [4,28]. Blood cultures should therefore be obtained before antimicrobial therapy whenever melioidosis is clinically suspected. However, localized disease may occur in the absence of bacteremia, emphasizing the importance of obtaining cultures from abscesses, skin lesions, lymph nodes, or other affected tissues. Limited laboratory capacity and delayed organism identification remain major barriers to diagnosis in many endemic regions, contributing to under-recognition of disease and delayed initiation of effective antimicrobial therapy [1,2].

The pooled mortality estimate of 12% demonstrates that pediatric melioidosis remains associated with substantial mortality despite advances in antimicrobial therapy. Mortality varied considerably across studies, reflecting differences in disease severity, referral pathways, access to healthcare, diagnostic capacity, intensive care availability, and underlying host characteristics. Improved clinical recognition and treatment have been associated with reduced mortality in Cambodia [8], whereas referral centers managing critically ill children with bacteremia and septic shock continue to report high case-fatality rates [28]. In addition, thalassemia major has been identified as an important risk factor for severe pediatric melioidosis in Malaysia, highlighting the influence of host susceptibility on disease outcome [14].

Significant country-level differences were observed for localized infection, disseminated disease, pneumonia, bacteremia, and mortality. These findings likely reflect differences in environmental exposure, rainfall, bacterial ecology, healthcare infrastructure, diagnostic practices, referral systems, and surveillance rather than intrinsic biological differences between populations. Recent reports from Vietnam, Brazil, Indonesia, and India further indicate that pediatric melioidosis remains underrecognized in many regions where environmental conditions support the presence of *B. pseudomallei* [12,17,23,30]. These observations are consistent with global modelling studies suggesting that the true burden of melioidosis is substantially underestimated [3]. Geographic variation may also partly reflect strain-level heterogeneity in *B. pseudomallei*. The organism has a highly diverse genome, and variable virulence determinants have been associated with specific clinical phenotypes. In Australian isolates, the *B. mallei*-like bimA allele (bimA_Bm_) has been strongly associated with neurological melioidosis, offering a biologically plausible explanation for the relative overrepresentation of neuromelioidosis reported in Australian cohorts compared with many Southeast Asian pediatric series [32,33]. Conversely, the near absence of pediatric parotitis in Australia may reflect differences in both exposure pathways and circulating bacterial populations. These hypotheses cannot be tested directly in the present meta-analysis because genotype or strain data were rarely reported in the included pediatric studies.

This review has several strengths. It represents the first meta-analysis specifically designed to quantify the prevalence of major clinical manifestations, organ involvement, bacteremia, and mortality among children with melioidosis. The inclusion of studies from multiple endemic regions provides a broader overview of disease presentation than individual institutional reports, while the use of a prospectively registered protocol, independent study selection, structured quality assessment, and random-effects meta-analysis strengthens methodological rigor. Furthermore, subgroup analyses according to country provide novel insights into geographical variation that have not been comprehensively evaluated previously.

Several limitations should also be considered. Most included studies were retrospective observational cohorts and were therefore susceptible to incomplete clinical documentation, referral bias, and inconsistent diagnostic evaluation. The risk-of-bias assessment indicated moderate or high overall risk in most studies, primarily because of uncertain representativeness, selected clinical cohorts, incomplete follow-up reporting, or diagnostic uncertainty. Although non-applicable NOS items were excluded from the overall judgment rather than interpreted as favorable assessments, these methodological limitations may affect the robustness of the pooled estimates and should be considered alongside the substantial statistical heterogeneity. Definitions of localized and disseminated disease varied across studies, and several uncommon manifestations were reported by relatively few studies, limiting the precision of pooled estimates. Diagnostic ascertainment was also heterogeneous; although most studies used culture confirmation, several historical or surveillance studies relied on mixed serological, epidemiological, or administrative criteria, potentially introducing case misclassification. Individual patient-level data were unavailable, precluding detailed analyses of important prognostic factors such as age, nutritional status, underlying diseases, antimicrobial therapy, bacteremia, and diagnostic delay. Mortality was not consistently reported according to disease extent or age group; therefore, reliable subgroup analyses comparing localized versus disseminated disease or neonates versus older children could not be performed. In addition, strain and virulence-genotype data were rarely available, preventing direct assessment of whether bacterial genetic variation contributed to the observed geographic differences. Finally, the available evidence was concentrated largely in Southeast Asia and northern Australia, with comparatively limited data from South Asia, Africa, and Latin America, restricting the global generalizability of the findings.

The findings of this review have several important implications for clinical practice and future research. Improved recognition of characteristic pediatric manifestations, particularly suppurative parotitis, skin and soft tissue infection, lymphadenitis, and recurrent abscess formation, may facilitate earlier diagnosis and timely initiation of appropriate antimicrobial therapy. Strengthening laboratory capacity for culture and identification of *B. pseudomallei*, together with greater access to diagnostic imaging for detecting visceral abscesses, should remain priorities in endemic settings. Future studies should focus on large prospective multicenter cohorts using standardized case definitions, uniform diagnostic protocols, and comprehensive reporting of clinical manifestations and outcomes. Individual participant data meta-analyses would further improve understanding of prognostic factors associated with disseminated disease, bacteremia, and mortality, while expanded surveillance in underrepresented endemic regions will be essential for defining the true global burden of pediatric melioidosis.

## 5. Conclusions

Pediatric melioidosis remains an underrecognized infectious disease, and the lack of quantitative evidence on its clinical spectrum has limited timely diagnosis and effective management. In this systematic review and meta-analysis of 25 studies involving 6874 children from 10 countries, we comprehensively characterized the prevalence of major clinical manifestations, organ involvement, and mortality using random-effects models. Localized infection predominated (69%), whereas disseminated disease occurred in 30% of children. Skin and soft tissue infection (31%), lymphadenitis (24%), parotitis (13%), and pneumonia (18%) were the most common manifestations, while bacteremia affected 23% of patients and the pooled case-fatality rate remained 12%. Significant geographic variation further highlights differences in epidemiology, healthcare access, and diagnostic capacity across endemic regions. These findings provide the first comprehensive quantitative evidence describing the clinical spectrum of pediatric melioidosis and reinforce that, despite its predominantly localized presentation, severe invasive disease remains an important cause of childhood morbidity and mortality. The results support earlier clinical recognition, improved microbiological investigation, and strengthened surveillance in endemic and resource-limited settings. These priorities align with Sustainable Development Goal 3 (Good Health and Well-being), particularly through efforts to improve early diagnosis, reduce preventable childhood morbidity and mortality, and strengthen infectious disease surveillance in endemic settings. Future prospective multicenter studies using standardized diagnostic criteria and harmonized reporting are needed to better define risk factors for severe disease, explain regional variation, and improve strategies for the prevention, diagnosis, and management of pediatric melioidosis.

## Figures and Tables

**Table 1:** Characteristics of included studies.

Study [Ref]	Country	Study Design/Period	Pediatric Cases (*N*)	Age	Male, *n* (%)	Diagnostic Criteria
Chandna, 2021 [8]	Cambodia	Retrospective & prospective surveillance; 2009–2018	355	Median 5.7 y (IQR 3.1–9.5)	255 (71.8%)	Culture confirmed
Chandrakar, 2016 [9]	India	Prospective observational, mixed ages; 2012–2014	5	Neonate to <12 y	NR	Culture confirmed
Chanvitan, 2019 [10]	Thailand	Retrospective diagnostic cohort; 2002–2014	27	Median 8.4 y	NR	Culture and/or serology (study-defined)
Currie, 2021 [11]	Australia	Prospective observational cohort, mixed ages; 1989–2019	48	<15 y	NR	Culture confirmed
Dadheech, 2025 [12]	India	Ambispective cohort; 2020–Sep 2025	7	Median 5 y (range 2–16)	6 (85.7%)	Culture confirmed; Vitek 2/Vitek MS
Dance, 1989 [13]	Thailand	Prospective; 1986–1987	21	≤11 y	NR	Culture-positive melioidosis
Fong, 2015 [14]	Malaysia	Retrospective cohort; 2001–2012	27	Median approximately 7 y	NR	Culture confirmed
Foong, 2015 [15]	Singapore	Retrospective cohort; 2002–Jan 2014	17	Median 12.5 y (range 2–15)	NR	Culture confirmed
Heng, 1998 [16]	Singapore	National surveillance, mixed ages; 1989–1996	9	0–14 y	NR	Culture or presumptive IHA under surveillance definition
Lima, 2021 [17]	Brazil	Retrospective surveillance; 2005–2019	10	Median 11 y (range 3–14)	NR	Confirmed/suspected cases under state surveillance definitions
Lumbiganon, 1995 [18]	Thailand	Retrospective pediatric cohort; 1979–1993	55	Localized median 6 y; disseminated median 11 y	NR	Culture confirmed
Lumbiganon, 2011 [19]	Thailand	Retrospective cohort with prospective follow-up; 1994–2006	37	Range 1 month–15 y	NR	Culture-confirmed localized melioidosis
McLeod, 2015 [4]	Australia	Prospective pediatric cohort; 1989–2013	45	7 months–16 y	NR	Culture confirmed
Mohan, 2020 [20]	Malaysia	Retrospective imaging cohort; 2014–2018	9	<12 y	6 (66.7%)	Culture confirmed
Mohan, 2021 [21]	Malaysia	Retrospective descriptive subgroup; 2011–2020	20	Median reported in full text; <12 y	9 (45.0%)	Culture confirmed
Pagnarith, 2010 [22]	Cambodia	Retrospective laboratory-based cohort; 2005–2008	39	Median 7.8 y (range 1.6–16.2)	NR	Culture confirmed
Pham, 2024 [23]	Vietnam	Retrospective observational cohort; Jul 2015–Aug 2019	35	Median 6 y (IQR 3–8)	NR	Culture confirmed with molecular confirmation
Pongrithsukda, 1988 [24]	Thailand	Retrospective pediatric cohort; NR	18	Mean 6.8 y (range 8 months–15 y)	11 (61.1%)	Culture (12/18) or historical IHA-based criteria
Sam, 2006 [25]	Malaysia	Retrospective pediatric cohort; 1976–2005	16	Mean 9.7 y (range 19 days–15 y)	13 (81.3%)	Culture confirmed
The Lao Melioidosis Research Group, 2025 [26]	Laos	Prospective hospital-based cohort, pediatric subgroup; 1999–2020	385	Median 6 y (IQR 4–9)	202 (52.5%)	Culture confirmed
Sitthikarnkha, 2025 [27]	Thailand	Retrospective national administrative database; 2015–2023	5044	Age categories; <18 y	2941 (58.3%)	Principal ICD-coded melioidosis diagnosis
Smith, 2017 [28]	Australia	Retrospective pediatric subgroup; 1998–Mar 2017	10	Range 3 days–14 y	NR	Culture confirmed
Suy, 2026 [29]	Cambodia	Ambispective diagnostic cohort; Jul 2022–Dec 2024	56	Retrospective median 5.4 y; prospective median 3.9 y	NR	Culture confirmed; AMD-RDT evaluated prospectively
Tauran, 2018 [30]	Indonesia	Retrospective multicentre case compilation; 2012–2017	15	Range 1 day–11 y	NR	Culture confirmed
Turner, 2016 [31]	Cambodia	Retrospective pediatric cohort; 2009–2013	173	Median 5.7 y (range 8 days–15.9 y)	NR	Culture confirmed

AMD-RDT, Active Melioidosis Detect Plus Rapid Diagnostic Test; ICD, International Classification of Diseases; IHA, indirect hemagglutination; IQR, interquartile range; MS, mass spectrometry; NR, not reported; y, year; Diagnostic note: Culture isolation of *B. pseudomallei* was considered the reference standard. Studies using mixed serological, surveillance, or administrative definitions are identified explicitly because these approaches carry greater diagnostic uncertainty than culture confirmation.

**Table 2:** Clinical manifestations and mortality reported in pediatric melioidosis studies.

Study [Ref]	Localized	Disseminated	Parotitis	Lymphadenitis	Skin/SSTI	Pneumonia	Neurological	Hepatic	Splenic	Bacteremia	Septic shock	Mortality
Chandna, 2021 [8]	NR	NR	97/355 (27.3)	58/355 (16.3)	96/355 (27.0)	69/355 (19.4)	NR	NR	NR	73/355 (20.6)	NR	41/355 (11.5)
Chandrakar, 2016 [9]	2/5 (40.0)	3/5 (60.0)	NR	NR	NR	3/5 (60.0)	NR	NR	NR	NR	NR	1/5 (20.0)
Chanvitan, 2019 [10]	19/27 (70.4)	8/27 (29.6)	4/27 (14.8)	NR	9/27 (33.3)	7/27 (25.9)	NR	8/27 (29.6)	6/27 (22.2)	NR	NR	1/27 (3.7)
Currie, 2021 [11]	NR	NR	NR	NR	NR	NR	NR	NR	NR	NR	NR	3/48 (6.2)
Dadheech, 2025 [12]	5/7 (71.4)	2/7 (28.6)	0/7 (0.0)	1/7 (14.3)	2/7 (28.6)	3/7 (42.9)	1/7 (14.3)	2/7 (28.6)	NR	NR	NR	2/7 (28.6)
Dance, 1989 [13]	20/21 (95.2)	1/21 (4.8)	8/21 (38.1)	NR	NR	NR	NR	NR	NR	NR	NR	1/21 (4.8)
Fong, 2015 [14]	7/27 (25.9)	20/27 (74.1)	0/27 (0.0)	NR	NR	NR	NR	NR	NR	20/27 (74.1)	NR	16/27 (59.3)
Foong, 2015 [15]	14/17 (82.4)	3/17 (17.6)	0/17 (0.0)	NR	9/17 (52.9)	NR	NR	NR	NR	3/17 (17.6)	2/17 (11.8)	2/17 (11.8)
Heng, 1998 [16]	NR	NR	NR	NR	NR	NR	NR	NR	NR	NR	NR	0/9 (0.0)
Lima, 2021 [17]	NR	NR	NR	NR	NR	9/10 (90.0)	2/10 (20.0)	NR	NR	NR	8/10 (80.0)	6/10 (60.0)
Lumbiganon, 1995 [18]	35/55 (63.6)	20/55 (36.4)	14/55 (25.5)	NR	NR	NR	NR	NR	NR	NR	NR	12/55 (21.8)
Lumbiganon, 2011 [19]	37/37 (100.0)	0/37 (0.0)	11/37 (29.7)	NR	14/37 (37.8)	NR	NR	NR	NR	0/37 (0.0)	0/37 (0.0)	0/37 (0.0)
McLeod, 2015 [4]	37/45 (82.2)	8/45 (17.8)	0/45 (0.0)	NR	27/45 (60.0)	9/45 (20.0)	3/45 (6.7)	NR	NR	7/45 (15.6)	NR	3/45 (6.7)
Mohan, 2020 [20]	NR	NR	NR	NR	NR	2/9 (22.2)	NR	1/9 (11.1)	9/9 (100.0)	NR	NR	2/9 (22.2)
Mohan, 2021 [21]	NR	NR	NR	19/20 (95.0)	1/20 (5.0)	1/20 (5.0)	NR	NR	NR	3/20 (15.0)	NR	1/20 (5.0)
Pagnarith, 2010 [22]	27/39 (69.2)	12/39 (30.8)	15/39 (38.5)	4/39 (10.3)	7/39 (17.9)	3/39 (7.7)	1/39 (2.6)	NR	NR	6/39 (15.4)	NR	8/39 (20.5)
Pham, 2024 [23]	21/35 (60.0)	14/35 (40.0)	15/35 (42.9)	NR	NR	10/35 (28.6)	NR	NR	NR	7/35 (20.0)	NR	4/35 (11.4)
Pongrithsukda, 1988 [24]	12/18 (66.7)	6/18 (33.3)	3/18 (16.7)	NR	NR	6/18 (33.3)	NR	NR	NR	NR	NR	3/18 (16.7)
Sam, 2006 [25]	9/16 (56.2)	7/16 (43.8)	0/16 (0.0)	NR	6/16 (37.5)	NR	NR	NR	NR	7/16 (43.8)	NR	4/16 (25.0)
The Lao Melioidosis Research Group, 2025 [26]	303/385 (78.7)	82/385 (21.3)	NR	NR	NR	NR	NR	NR	NR	59/385 (15.3)	NR	32/347 (9.2)
Sitthikarnkha, 2025 [27]	NR	NR	NR	NR	NR	888/5044 (17.6)	NR	NR	NR	NR	146/5044 (2.9)	84/5044 (1.7)
Smith, 2017 [28]	4/10 (40.0)	6/10 (60.0)	0/10 (0.0)	0/10 (0.0)	3/10 (30.0)	2/10 (20.0)	2/10 (20.0)	0/10 (0.0)	NR	6/10 (60.0)	NR	5/10 (50.0)
Suy, 2026 [29]	NR	NR	18/56 (32.1)	25/56 (44.6)	13/56 (23.2)	NR	NR	NR	NR	2/56 (3.6)	NR	0/56 (0.0)
Tauran, 2018 [30]	1/15 (6.7)	14/15 (93.3)	NR	NR	NR	3/15 (20.0)	0/15 (0.0)	NR	NR	14/15 (93.3)	NR	7/15 (46.7)
Turner, 2016 [31]	131/173 (75.7)	42/173 (24.3)	51/173 (29.5)	NR	60/173 (34.7)	33/173 (19.1)	NR	NR	NR	39/173 (22.5)	NR	29/173 (16.8)

NR indicates that the outcome was not reported or was not separately extractable; Values are n/N (%); SSTI, skin and soft tissue infection.

**Table 3:** Geographic variation in reported pediatric melioidosis presentations across included studies.

Country	Localized	Disseminated	Parotitis	Neurological	Bacteremia	Mortality
Thailand	63.6–100.0%	4.8–36.4%	14.8–38.1%	NR	NR	1.7–21.8%
Cambodia	69.2–75.7%	24.3–30.8%	27.3–38.5%	2.6%	3.6–22.5%	11.5–20.5%
Malaysia	25.9–56.2%	43.8–74.1%	0.0%	NR	15.0–74.1%	5.0–59.3%
Australia	40.0–82.2%	17.8–60.0%	0.0%	6.7–20.0%	15.6–60.0%	6.2–50.0%
India	40.0–71.4%	28.6–60.0%	0.0%	14.3%	NR	20.0–28.6%
Singapore	82.4%	17.6%	0.0%	NR	17.6%	11.8%
Brazil	NR	NR	NR	20.0%	NR	60.0%
Indonesia	6.7%	93.3%	NR	NR	93.3%	46.7%
Laos	78.7%	21.3%	NR	NR	15.3%	9.2%
Vietnam	60.0%	40.0%	42.9%	NR	20.0%	11.4%

Values are ranges of study-specific percentages among studies reporting each outcome; NR, not reported or not separately extractable. These descriptive ranges are not pooled country-level estimates. Key patterns include frequent parotitis in several Southeast Asian cohorts, absence of parotitis in the contributing Australian pediatric cohorts, relatively prominent neurological involvement in Australian series, and higher disseminated disease, bacteremia, and mortality in some referral cohorts from Malaysia and Indonesia.

## Data Availability

No new data were created or analyzed in this study. Data sharing is not applicable to this article.

## Supplementary Materials

The following supporting information can be downloaded at: https://www.mdpi.com/article/10.3390/life16091555/s1, Supplementary File S1: Search strategies; Supplementary Table S1: Quality of studies by Newcastle–Ottawa Scale (NOS); Supplementary Figure S1: Pooled prevalence of clinical manifestations and organ involvement in pediatric melioidosis; Supplementary Figure S2: Country subgroup analyses; Supplementary Figure S3: Funnel plots.

## Abbreviations

The following abbreviations are used in this manuscript:

CI	confidence interval
ICD	international classification of diseases
IHA	indirect hemagglutination
IQR	interquartile range
MS	mass spectrometry
SSSI	skin and soft tissue infection
