# Supplementary material for: Clinical Spectrum and Organ Involvement in Pediatric Melioidosis: A Systematic Review and Meta-Analysis
Source: Life (Basel). 2026 Sep 16;16(9):1555. doi: 10.3390/life16091555 (PMC13608709; doi:10.3390/life16091555)
Supplement: Supplementary file 1 [file life-16-01555-s001.zip › Supplementary Figure S2_Country subgroup analyses - fig-revised.pdf]

Clinical Spectrum and Organ Involvement in Pediatric Melioidosis: A Systematic Review and Meta-Analysis

Jongkonnee Thanasai [1], Anchalee Chittamma [2], Supphachoke Khemla [3], Atthaphong Phongphithakchai [4], Moragot Chatatikun [5], Jitbanjong Tangpong [5,], Sa-ngob Laklaeng [5], Jirarat Songsri[5] and Wiyada Kwanhian Klangbud [6,7,*]

[1] Faculty of Medicine, Mahasarakham University, Mahasarakham 44000, Thailand; jongkonnee@msu.ac.th

[2] Department of Pathology, Faculty of Medicine Ramathibodi Hospital, Mahidol University, Bangkok 10400, Thailand; anchalee.chi@mahidol.ac.th

[3] Division of Infectious Diseases, Department of Internal Medicine, Nakhon Phanom Hospital, Nakhon Phanom 48000, Thailand; sup.mednkp@gmail.com

[4] Nephrology Unit, Division of Internal Medicine, Faculty of Medicine, Prince of Songkla University, Songkhla 90110, Thailand; atthaphong.p@psu.ac.th

[5] School of Allied Health Sciences, Walailak University, Nakhon Si Thammarat 80160, Thailand; moragot.ch@wu.ac.th (M.C.); rjitbanj@wu.ac.th (J.T.); sangob.ll@wu.ac.th (S.-n.L.)

[6] Medical Technology Program, Faculty of Science, Nakhon Phanom University, Nakhon Phanom 48000, Thailand

[7] Faculty of Medicine, Nakhon Phanom University, Nakhon Phanom 48000, Thailand

* Correspondence: wiyadakwanhian@gmail.com

Supplementary Figure S2. Country subgroup analyses

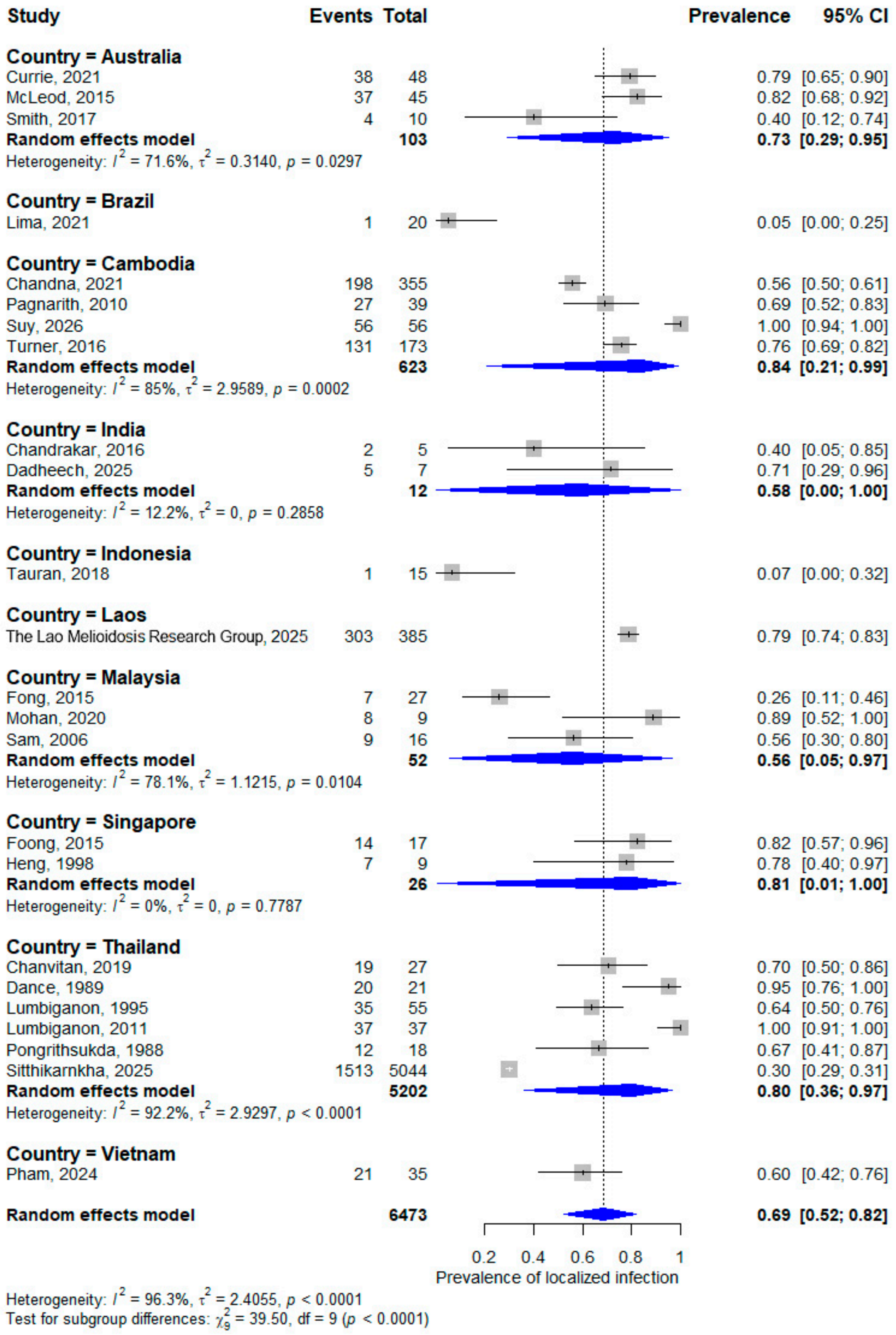

Supplementary Figure S2.1. Localized infection in different geographic variations

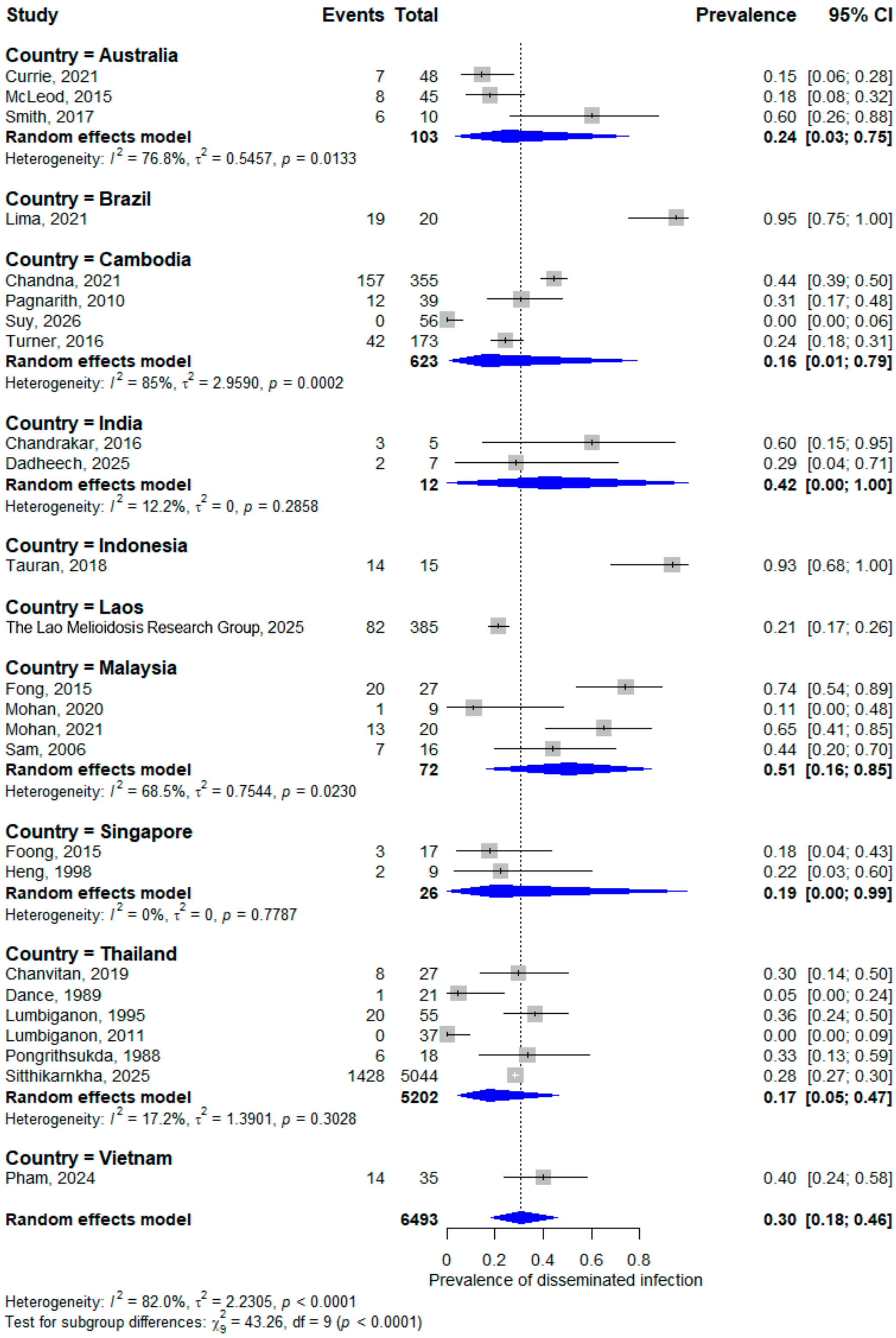

Supplementary Figure S2.2. Disseminated infection in different geographic variations

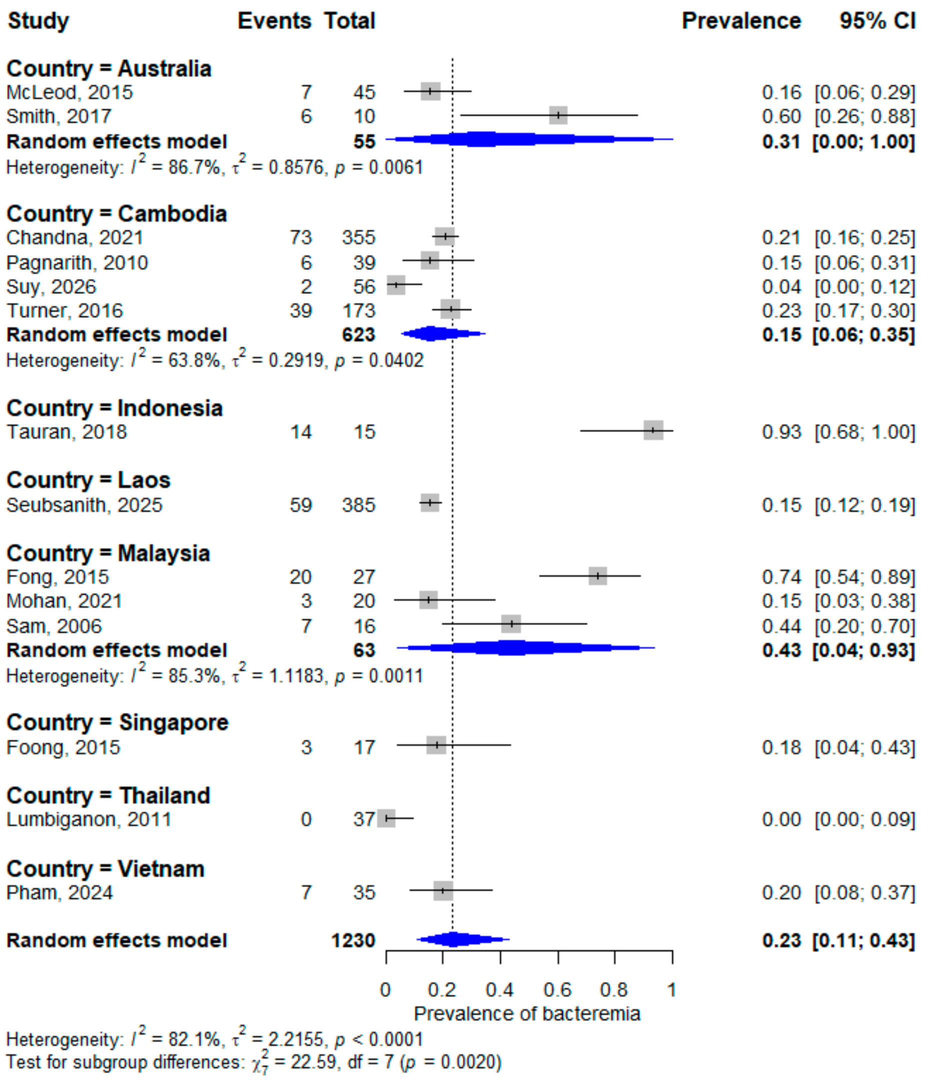

Supplementary Figure S2.3. Bacteremia in different geographic variations

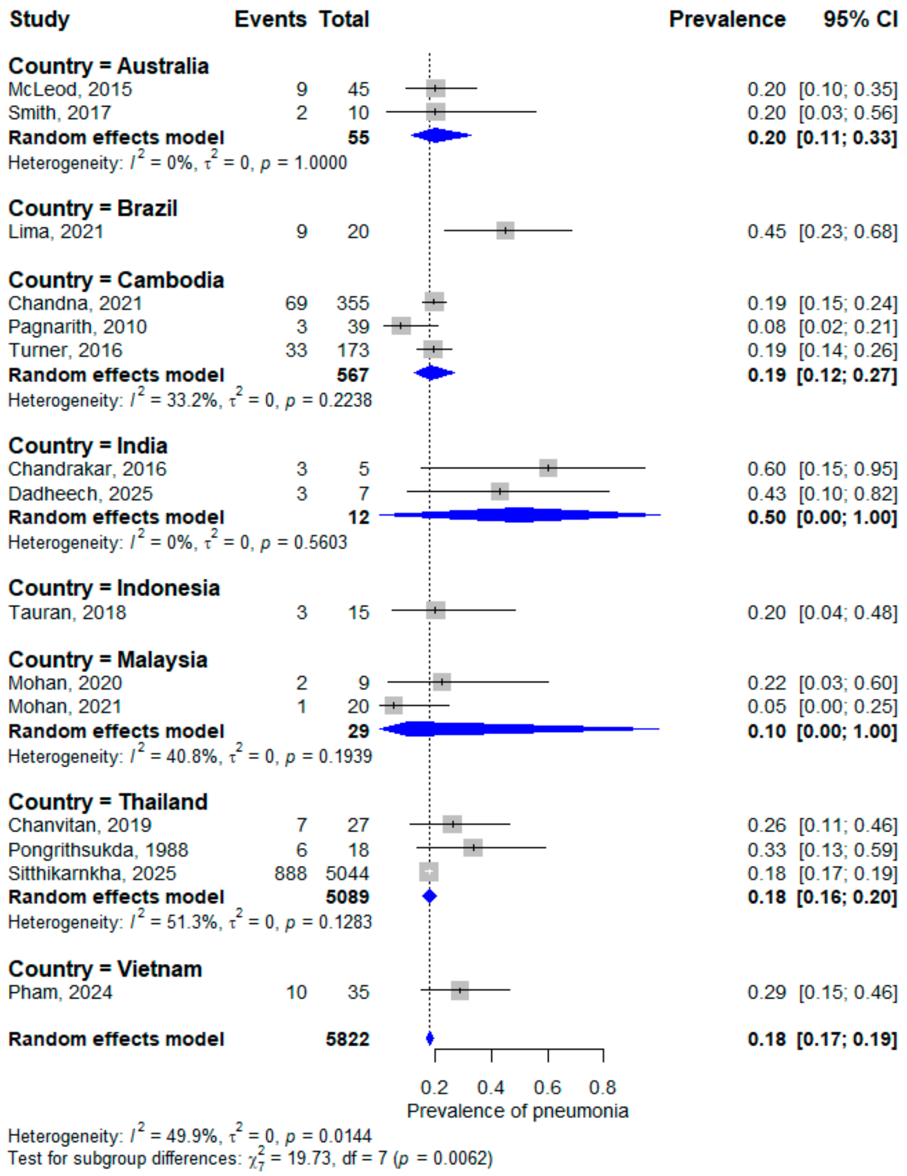

Supplementary Figure S2.4. Pneumonia in different geographic variations

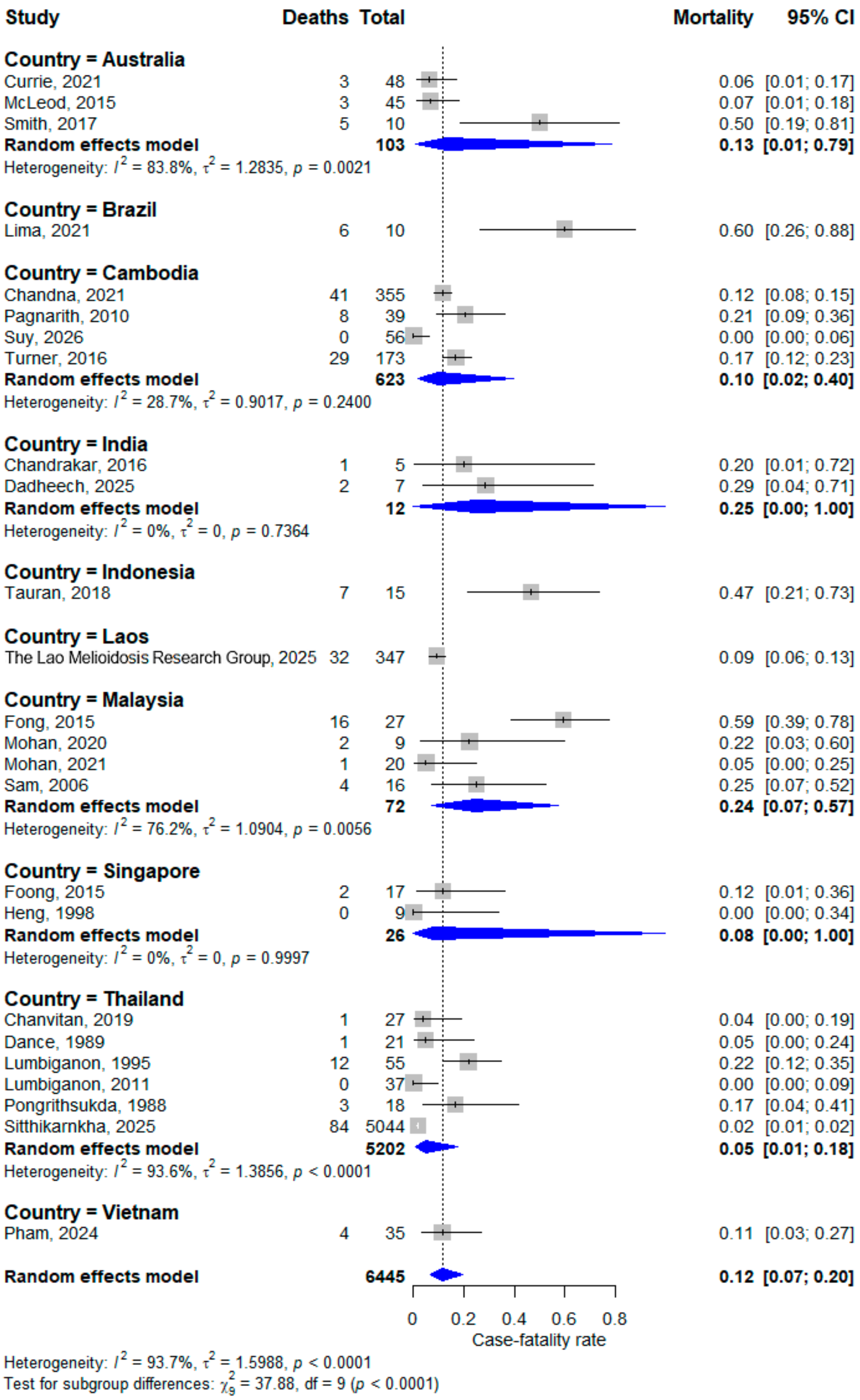

Supplementary Figure S2.5. Mortality rate in different geographic variations
