# Supplementary material for: Clinical Spectrum and Organ Involvement in Pediatric Melioidosis: A Systematic Review and Meta-Analysis
Source: Life (Basel). 2026 Sep 16;16(9):1555. doi: 10.3390/life16091555 (PMC13608709; doi:10.3390/life16091555)
Supplement: Supplementary file 1 [file life-16-01555-s001.zip › Supplementary Figure S3_funnel plot.pdf]

Clinical Spectrum and Organ Involvement in Pediatric Melioidosis: A Systematic Review and Meta-Analysis

Jongkonnee Thanasai [1], Anchalee Chittamma [2], Supphachoke Khemla [3], Atthaphong Phongphithakchai [4], Moragot Chatatikun [5], Jitbanjong Tangpong [5], Sa-ngob Laklaeng [5], Jirarat Songsri[5] and Wiyada Kwanhian Klangbud [6,7,*]

[1] Faculty of Medicine, Mahasarakham University, Mahasarakham 44000, Thailand; jongkonnee@msu.ac.th

[2] Department of Pathology, Faculty of Medicine Ramathibodi Hospital, Mahidol University, Bangkok 10400, Thailand; anchalee.chi@mahidol.ac.th

[3] Division of Infectious Diseases, Department of Internal Medicine, Nakhon Phanom Hospital, Nakhon Phanom 48000, Thailand; sup.mednkp@gmail.com

[4] Nephrology Unit, Division of Internal Medicine, Faculty of Medicine, Prince of Songkla University, Songkhla 90110, Thailand; atthaphong.p@psu.ac.th

[5] School of Allied Health Sciences, Walailak University, Nakhon Si Thammarat 80160, Thailand; moragot.ch@wu.ac.th (M.C.); rjitbanj@wu.ac.th (J.T.); sangob.ll@wu.ac.th (S.-n.L.)

[6] Medical Technology Program, Faculty of Science, Nakhon Phanom University, Nakhon Phanom 48000, Thailand

[7] Faculty of Medicine, Nakhon Phanom University, Nakhon Phanom 48000, Thailand

* Correspondence: wiyadakwanhian@gmail.com

Supplementary Figure S3. Funnel plots

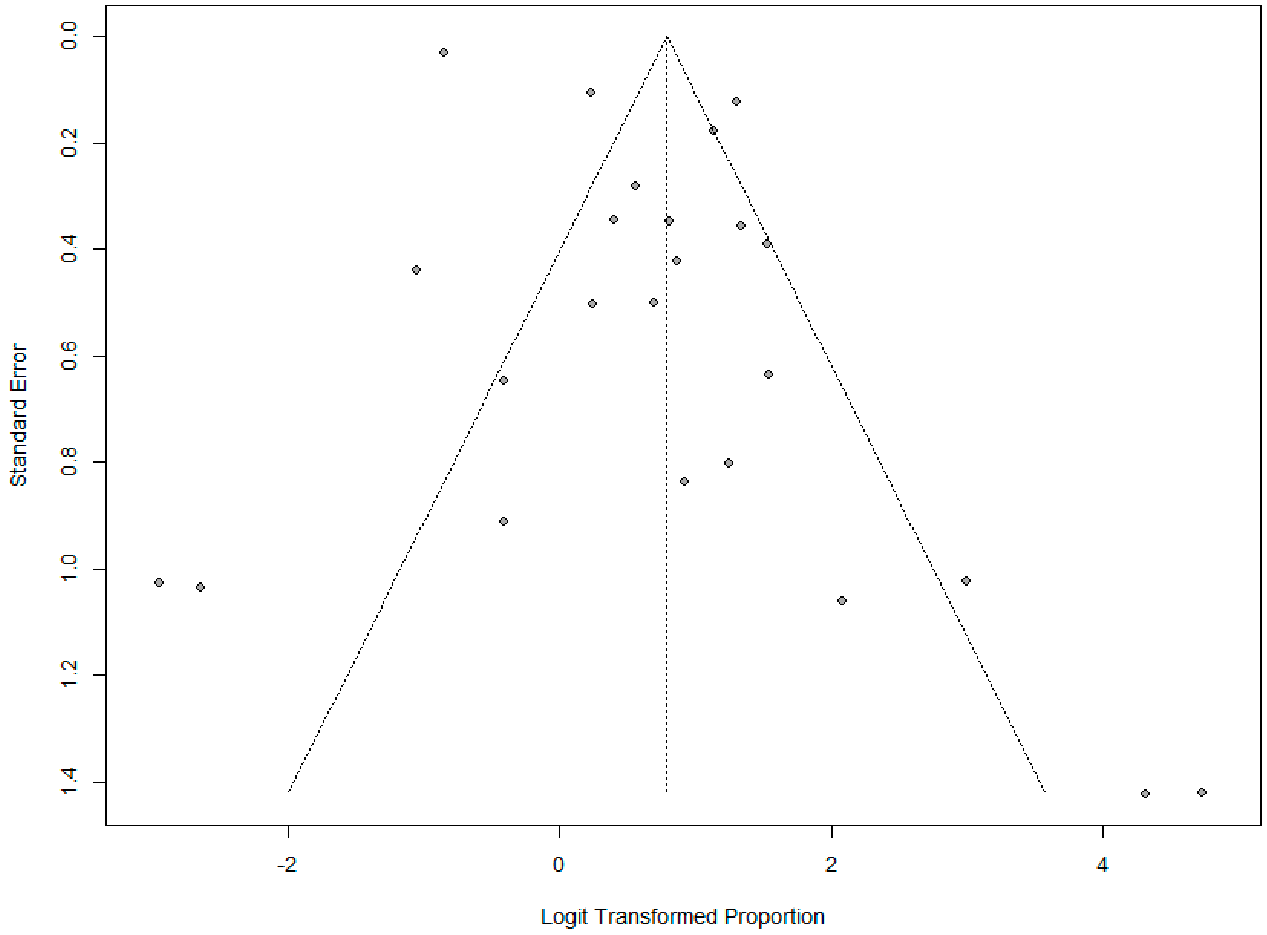

Supplementary Figure S3.1. Funnel plot of localized infection. Linear regression test of funnel plot asymmetry, t = 3.55, df = 22, *p*-value = 0.0018. Bias estimate: 3.6466 (SE = 1.0285), multiplicative residual heterogeneity variance tau^2 = 18.7697. predictor standard error and weight inverse variance.

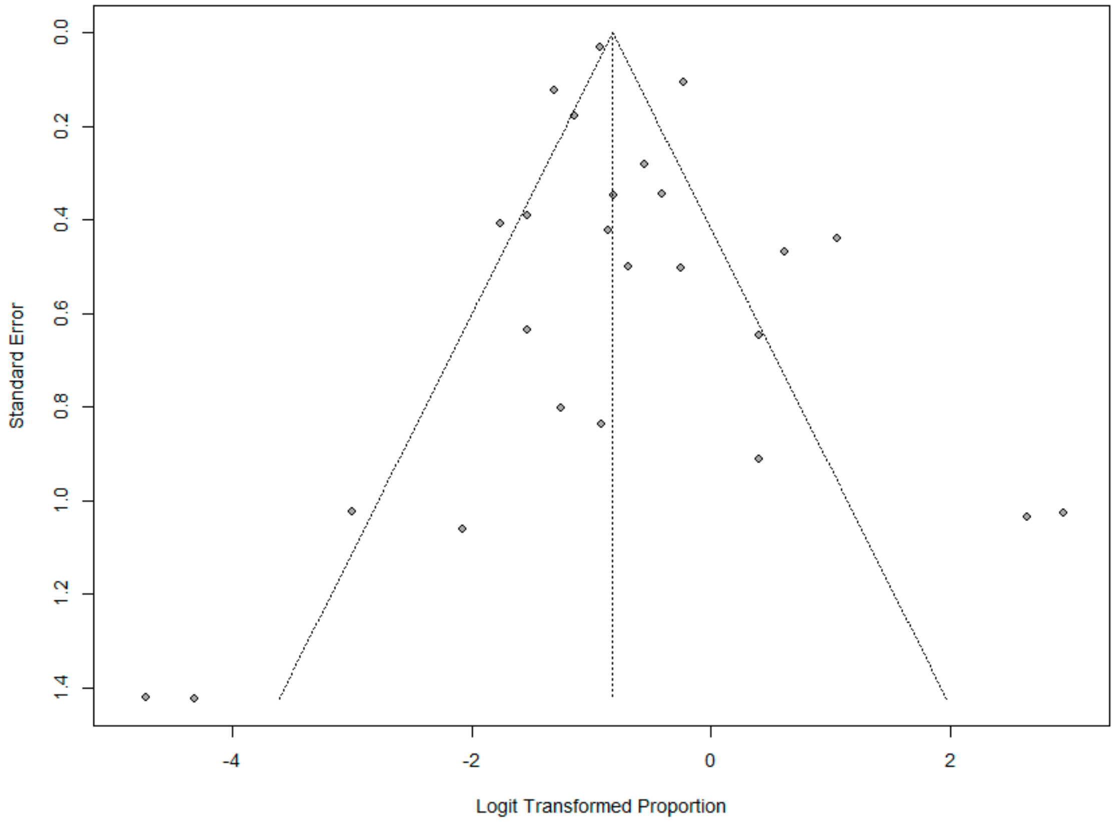

Supplementary Figure S3.2. Funnel plot of disseminated infection. Linear regression test of funnel plot asymmetry t = 0.84, df = 23, *p*-value = 0.4109, Bias estimate: 0.4852 (SE = 0.5793). Multiplicative residual heterogeneity variance ($tau^2 = 6.1788$), predictor standard error, and weight inverse variance.

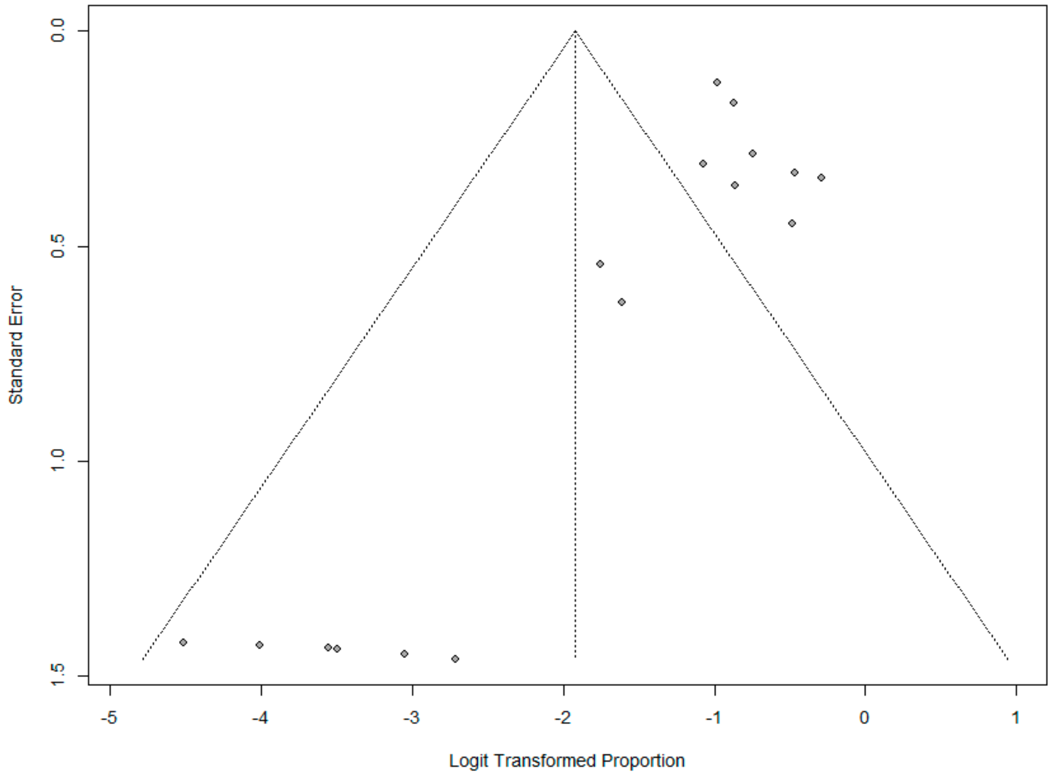

Supplementary Figure S3.3. Funnel plot of parotitis. Linear regression test of funnel plot asymmetry t =-2.90, df = 14, *p*-value = 0.0118, Bias estimate: -1.3431 (SE = 0.4639). Multiplicative residual heterogeneity variance (tau^2 = 6.1788), predictor standard error, and weight inverse variance.

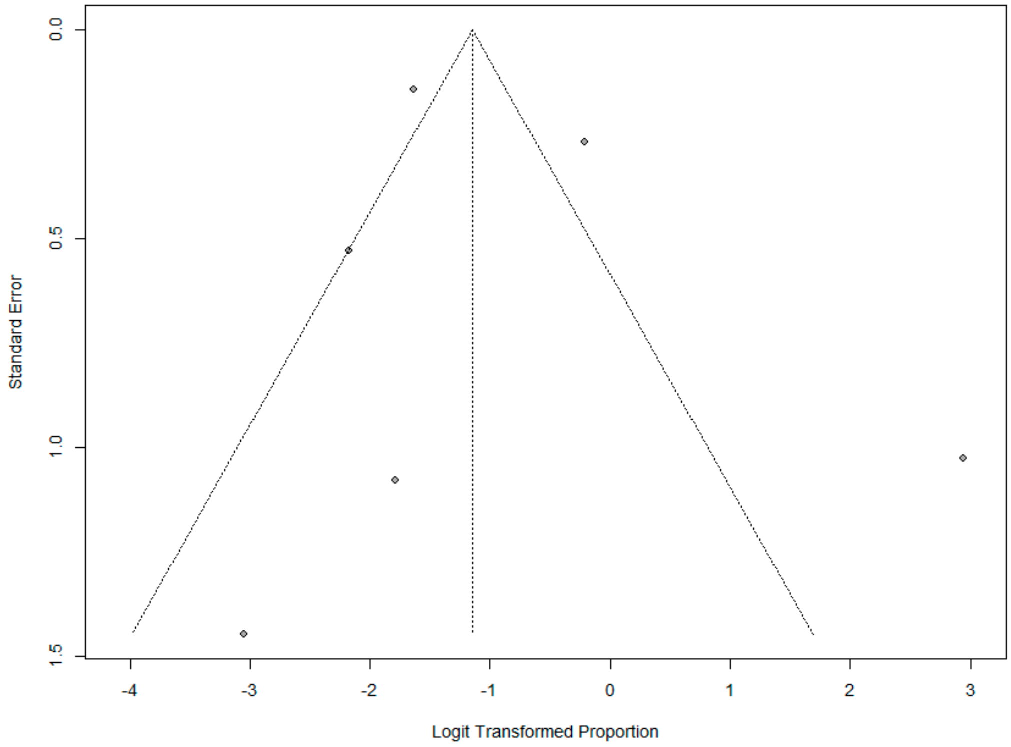

Supplementary Figure S3.4. Funnel plot of lymphadenitis. Number of studies (k=6) too small to test for small study effects (k.min=10).

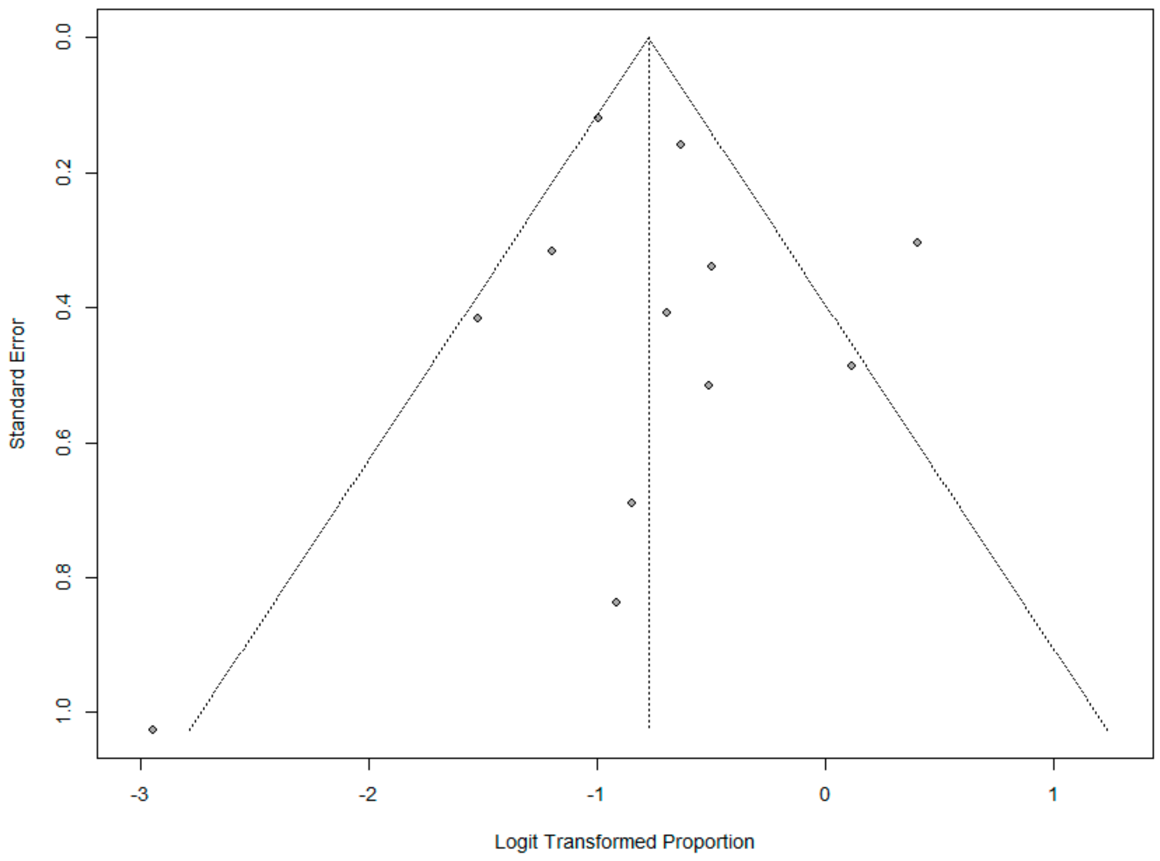

Supplementary Figure S3.5. Funnel plot of skin soft tissue infection (SSTI). Linear regression test of funnel plot asymmetry t = 0.24, df = 10, *p*-value = 0.8165, Bias estimate: 0.2204 (SE = 0.9253). Multiplicative residual heterogeneity variance ($tau^2 = 3.2787$), predictor standard error, and weight inverse variance.

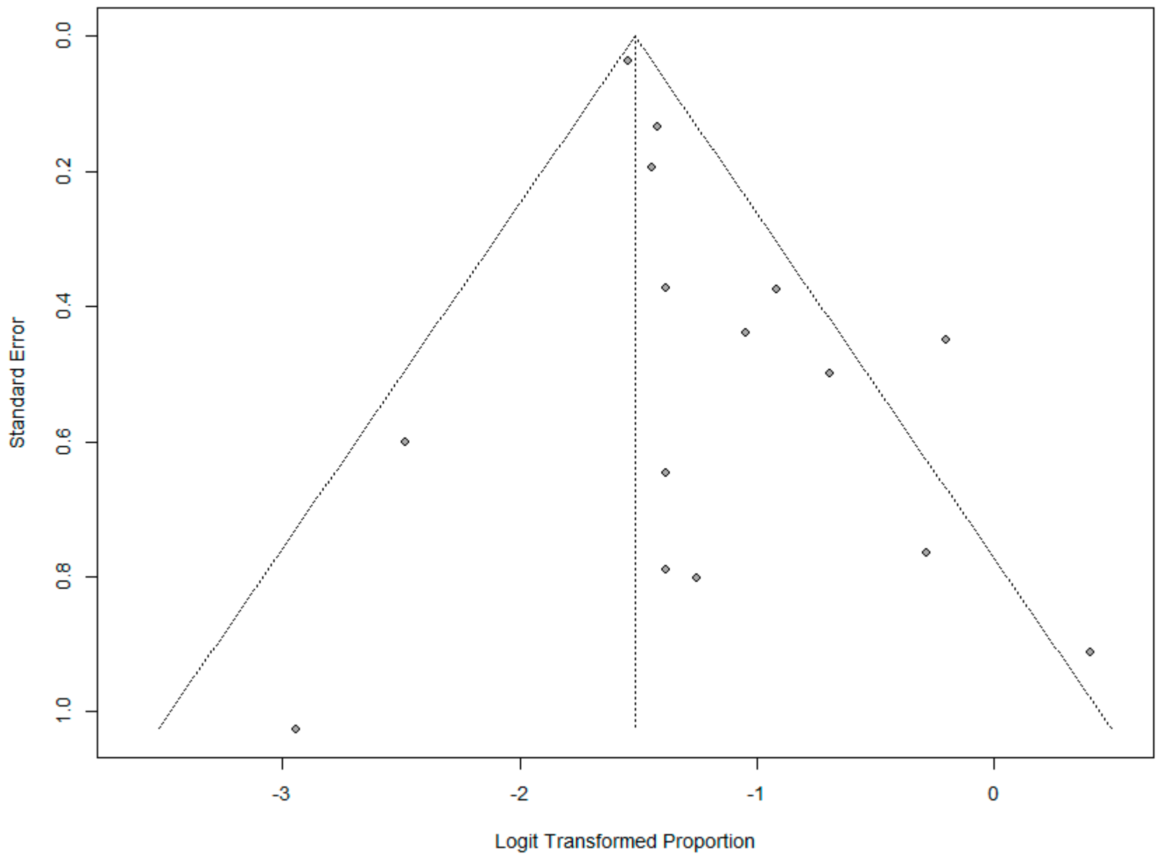

Supplementary Figure S3.6. Funnel plot of pneumonia. Linear regression test of funnel plot asymmetry t = 2.16, df = 13, *p*-value = 0.0497, Bias estimate: 0.8318 (SE = 0.3844). Multiplicative residual heterogeneity variance (tau^2 = 1.5808), predictor standard error, and weight inverse variance.

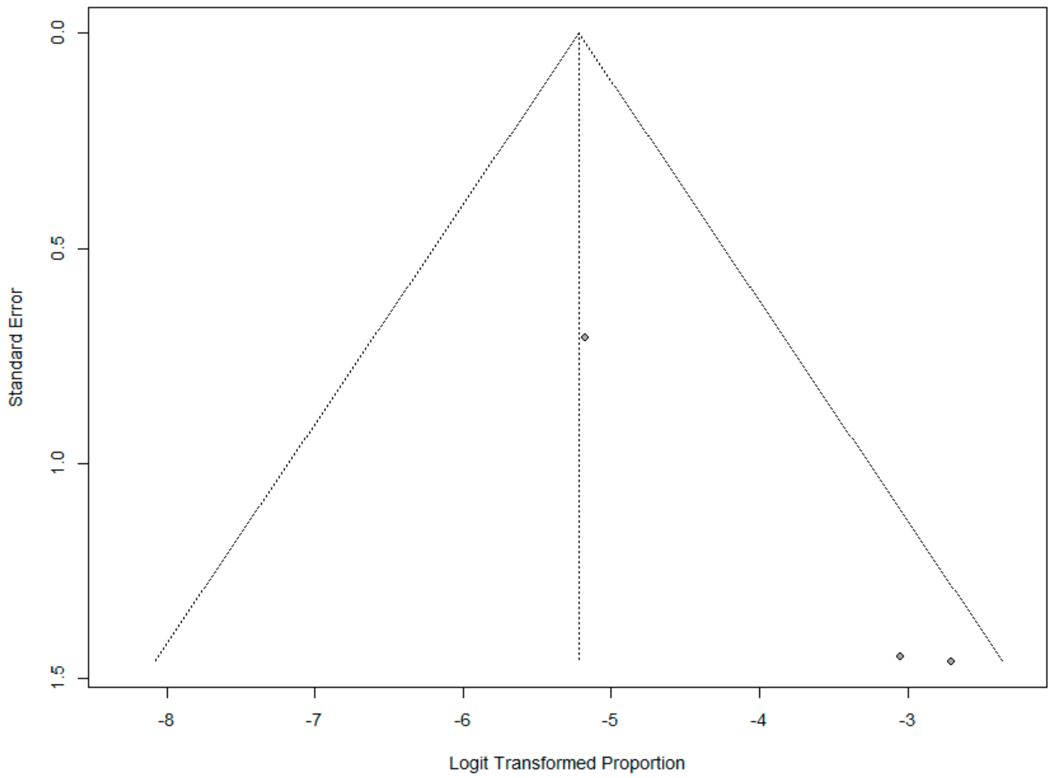

Supplementary Figure S3.7. Funnel plot of osteomyelitis. Number of studies (k=3) too small to test for small study effects (k.min=10).

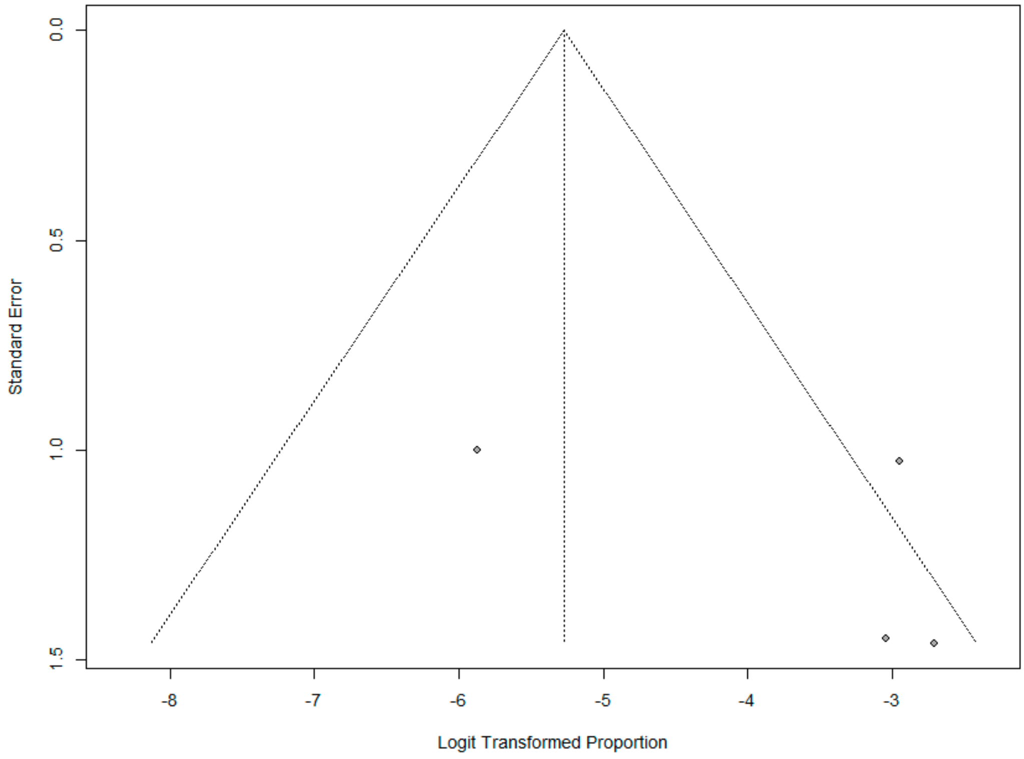

Supplementary Figure S3.8. Funnel plot of septic arthritis. Number of studies (k=4) too small to test for small study effects (k.min=10).

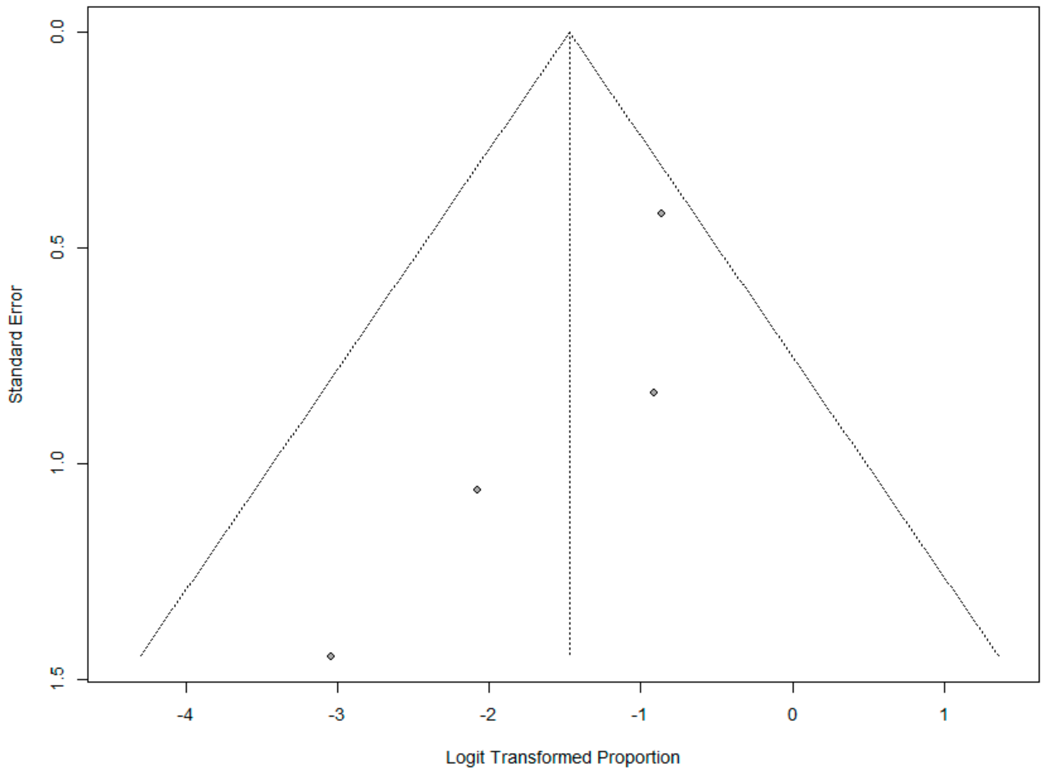

Supplementary Figure S3.9. Funnel plot of hepatic involvement. Number of studies (k=3) too small to test for small study effects (k.min=10).

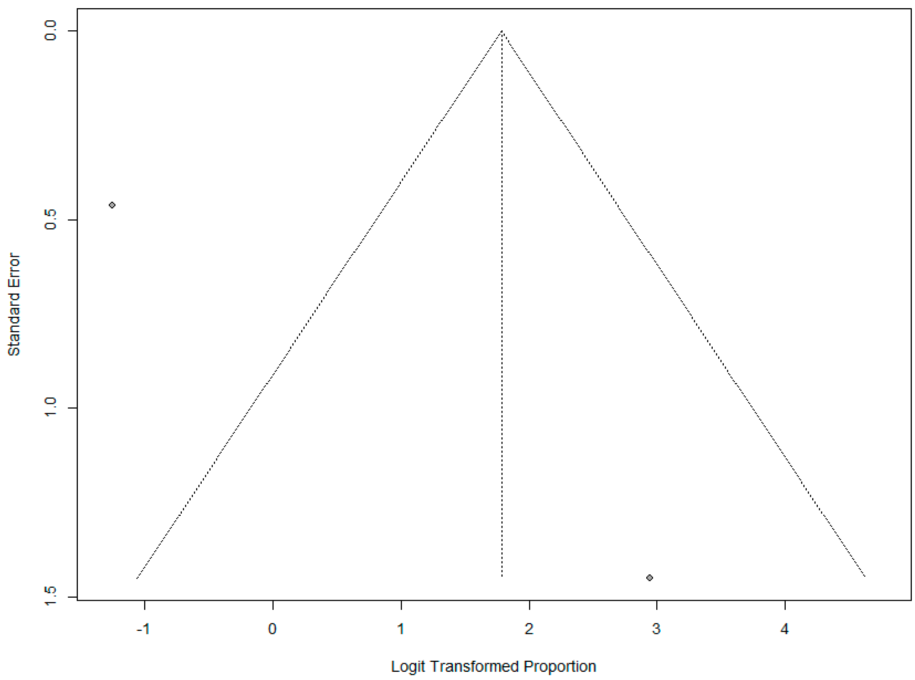

Supplementary Figure S3.10. Funnel plot of splenic involvement. Number of studies (k=3) too small to test for small study effects (k.min=10).

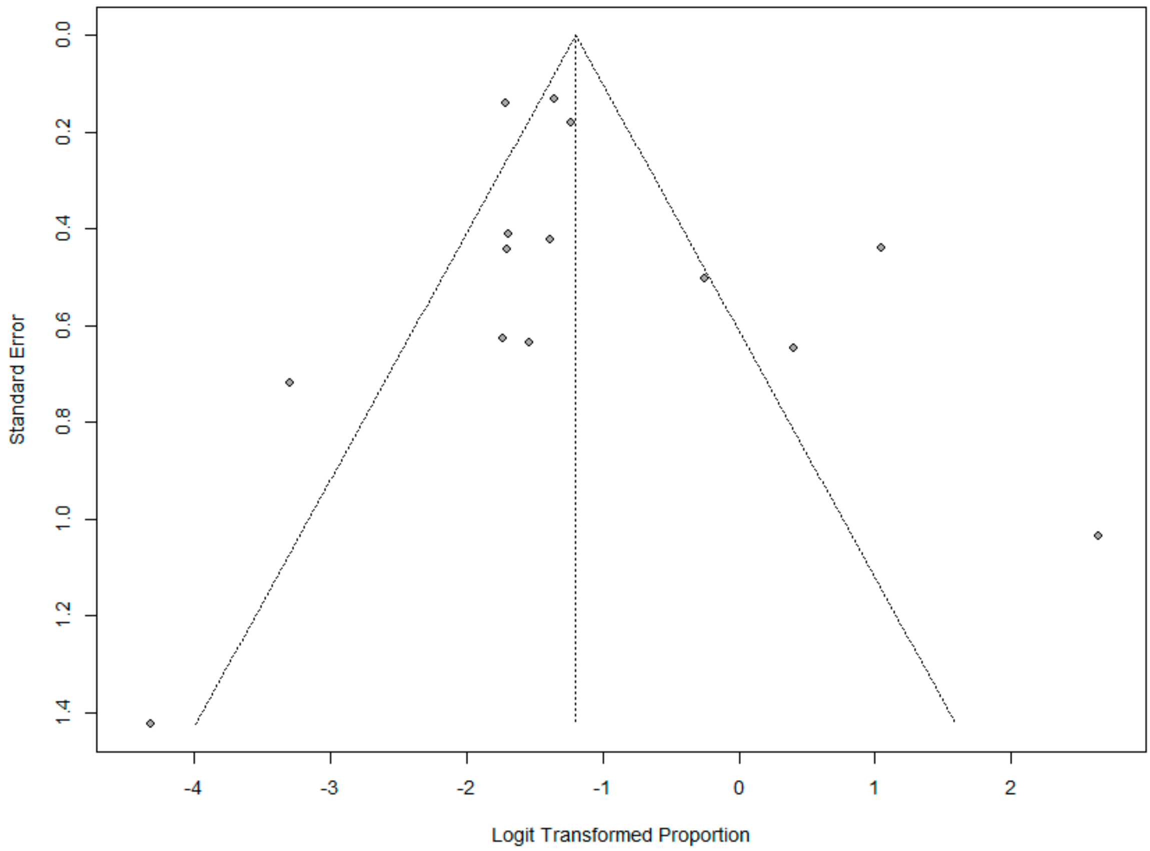

Supplementary Figure S3.11. Funnel plot of bacteremia. Linear regression test of funnel plot asymmetry t = 0.90, df = 12, *p*-value = 0.3875, Bias estimate: 0.9680 (SE = 1.0796). Multiplicative residual heterogeneity variance (tau^2 = 1.5808), predictor standard error, and weight inverse variance.

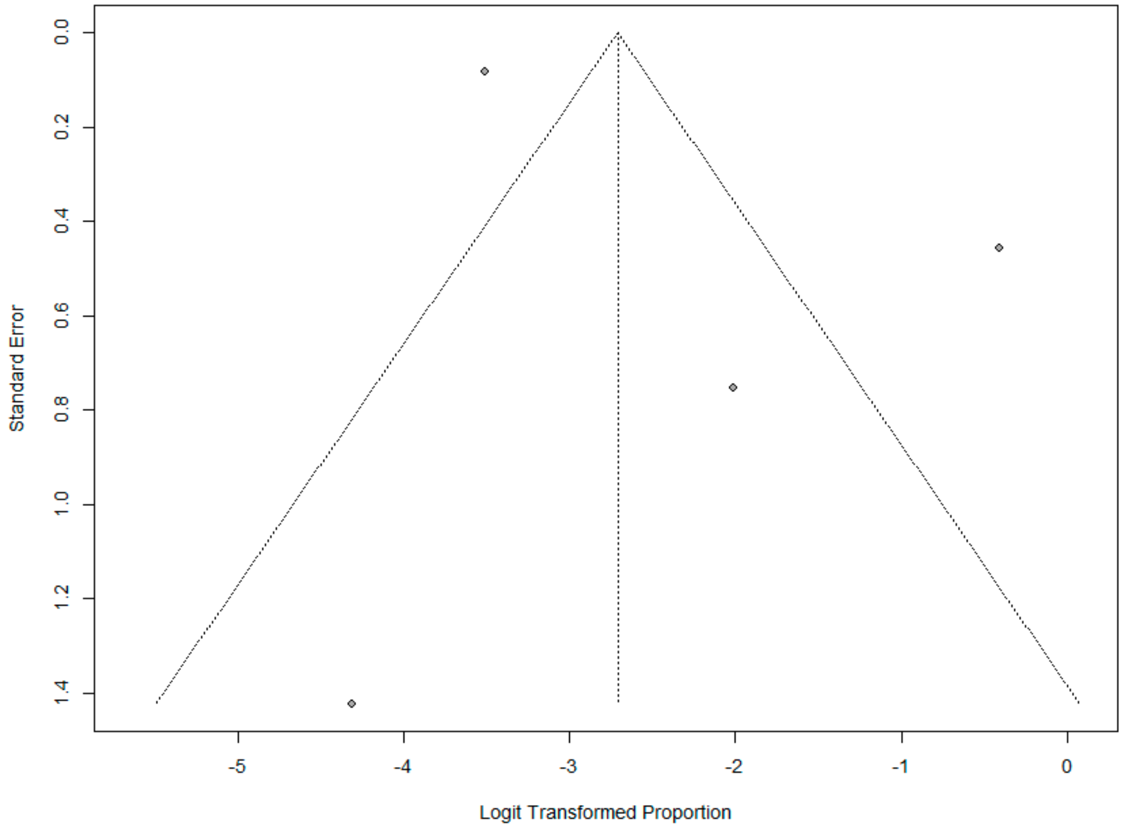

Supplementary Figure S3.12. Funnel plot of septic shock. Number of studies (k=3) too small to test for small study effects (k.min=10).

Mortality

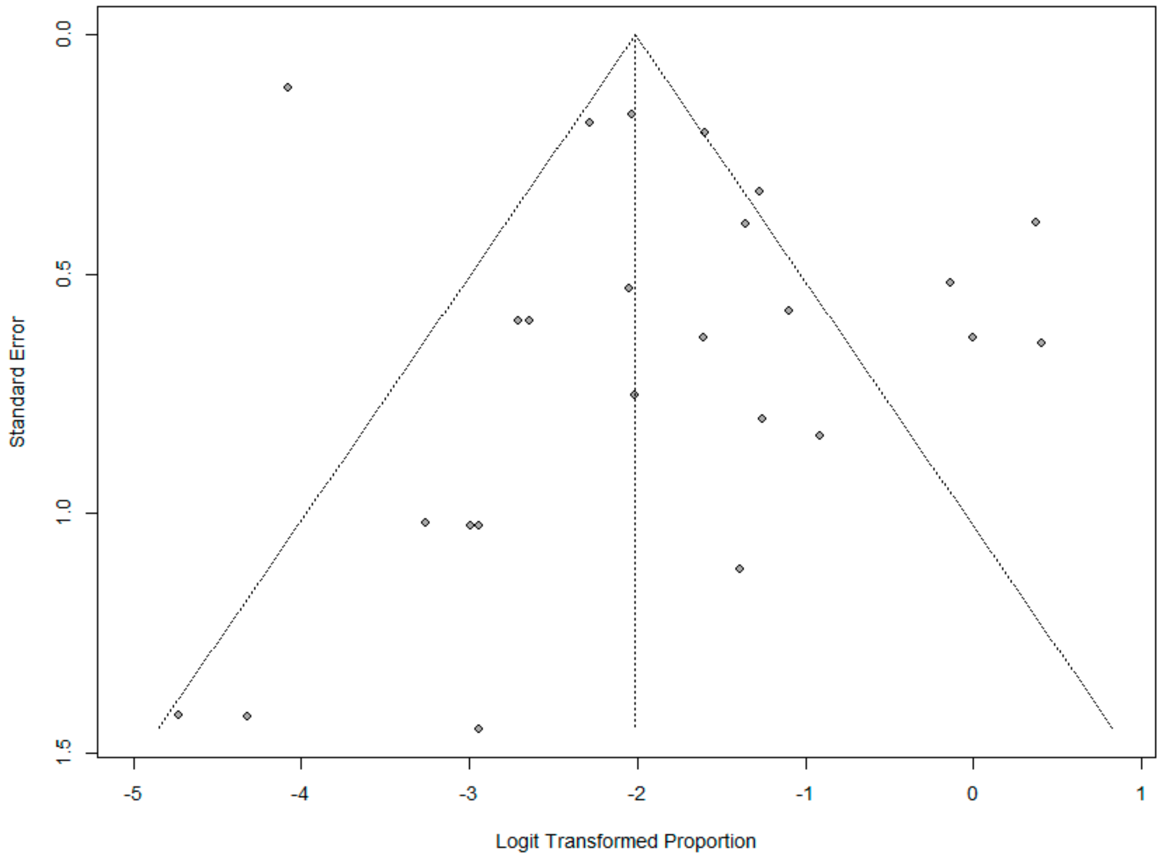

Supplementary Figure S3.13. Funnel plot of mortality rate. Linear regression test of funnel plot asymmetry t = 2.47, df = 23, *p*-value = 0.0213, Bias estimate: 2.7457 (SE = 1.1106). Multiplicative residual heterogeneity variance (tau^2 = 13.3099), predictor standard error, and weight inverse variance.
