# Supplementary material for: Clinical Spectrum and Organ Involvement in Pediatric Melioidosis: A Systematic Review and Meta-Analysis
Source: Life (Basel). 2026 Sep 16;16(9):1555. doi: 10.3390/life16091555 (PMC13608709; doi:10.3390/life16091555)
Supplement: Supplementary file 1 [file life-16-01555-s001.zip › Supplementary Table S1_NOS_revised.pdf]

Clinical Spectrum and Organ Involvement in Pediatric Melioidosis: A Systematic Review and Meta-Analysis

Jongkonnee Thanasai [1], Anchalee Chittamma [2], Supphachoke Khemla [3], Atthaphong Phongphithakchai [4], Moragot Chatatikun [5], Jitbanjong Tangpong [5], Sa-ngob Laklaeng [5], Jirarat Songsri[5] and Wiyada Kwanhian Klangbud [6,7,*]

1 Faculty of Medicine, Mahasarakham University, Mahasarakham 44000, Thailand; jongkonnee@msu.ac.th

2 Department of Pathology, Faculty of Medicine Ramathibodi Hospital, Mahidol University, Bangkok 10400, Thailand; anchalee.chi@mahidol.ac.th

3 Division of Infectious Diseases, Department of Internal Medicine, Nakhon Phanom Hospital, Nakhon Phanom 48000, Thailand; sup.mednkp@gmail.com

4 Nephrology Unit, Division of Internal Medicine, Faculty of Medicine, Prince of Songkla University, Songkhla 90110, Thailand; atthaphong.p@psu.ac.th

5 School of Allied Health Sciences, Walailak University, Nakhon Si Thammarat 80160, Thailand; moragot.ch@wu.ac.th (M.C.); rjitbanj@wu.ac.th (J.T.); sangob.ll@wu.ac.th (S.-n.L.)

6 Medical Technology Program, Faculty of Science, Nakhon Phanom University, Nakhon Phanom 48000, Thailand

7 Faculty of Medicine, Nakhon Phanom University, Nakhon Phanom 48000, Thailand

* Correspondence: wiyadakwanhian@gmail.com

Supplementary Table S1. Quality of studies by Newcastle–Ottawa Scale (NOS)

Study	S1 Representative cohort	S2 Non-exposed cohort	S3 Melioidosis ascertainment	S4 Outcome absent at start	C1/C2 Comparability	O1 Outcome ascertainment	O2 Follow-up duration	O3 Follow-up completeness	Key note	Overall risk
Chandna, 2021	Yes	N/A	Culture confirmed	N/A	N/A	Yes	NR/unclear	NR/unclear	Large surveillance cohort; follow-up items not assumed from outcome reporting alone.	Moderate
Chandrakar, 2016	Unclear	N/A	Culture confirmed	N/A	N/A	Yes	NR/unclear	NR/unclear	Only five pediatric cases; pediatric-specific treatment/follow-up incompletely stratified.	Moderate
Chanvitan, 2019	Unclear	N/A	Mixed culture/serology	N/A	N/A	Yes	NR/unclear	NR/unclear	Diagnostic ascertainment has potential misclassification from serologic component.	High
Currie, 2021	Yes	N/A	Culture confirmed	N/A	N/A	Yes	Adequate	Adequate/mostly complete	Prospective long-term cohort; pediatric subgroup outcome ascertainment reported.	Low
Dadheech, 2025	Unclear	N/A	Culture confirmed	N/A	N/A	Yes	NR/unclear	NR/unclear	Small ambispective cohort.	Moderate
Dance, 1989	Yes	N/A	Culture-positive series	N/A	N/A	Yes	NR/unclear	NR/unclear	Source rechecked; prospective culture-positive melioidosis series.	Moderate
Fong, 2015	Unclear	N/A	Culture confirmed	N/A	N/A	Yes	NR/unclear	NR/unclear	Retrospective referral cohort.	Moderate
Foong, 2015	Unclear	N/A	Culture confirmed	N/A	N/A	Yes	NR/unclear	NR/unclear	Retrospective cohort.	Moderate
Heng, 1998	Yes	N/A	Mixed culture/presumptive IHA	N/A	N/A	Yes	NR/unclear	NR/unclear	National surveillance; pediatric clinical details limited.	High

Study	S1 Representative cohort	S2 Non-exposed cohort	S3 Melioidosis ascertainment	S4 Outcome absent at start	C1/C2 Comparability	O1 Outcome ascertainment	O2 Follow-up duration	O3 Follow-up completeness	Key note	Overall risk
Lima, 2021	Unclear	N/A	Surveillance-confirmed/suspected framework	N/A	N/A	Yes	NR/unclear	NR/unclear	Case ascertainment based on surveillance definitions; diagnostic uncertainty acknowledged.	High
Lumbiganon, 1995	Yes	N/A	Culture confirmed	N/A	N/A	Yes	NR/unclear	NR/unclear	Retrospective pediatric cohort.	Moderate
Lumbiganon, 2011	Unclear	N/A	Culture confirmed	N/A	N/A	Yes	Adequate	Partly documented	Follow-up reported for 32/37; incomplete follow-up explicitly retained as limitation.	Moderate
McLeod, 2015	Yes	N/A	Culture confirmed	N/A	N/A	Yes	Adequate	Adequate/mostly complete	Prospective pediatric cohort.	Low
Mohan, 2020	No/selected	N/A	Culture confirmed	N/A	N/A	Yes	NR/unclear	NR/unclear	Imaging-selected cohort; limited representativeness for pooled organ prevalence.	High
Mohan, 2021	No/selected	N/A	Culture confirmed	N/A	N/A	Yes	NR/unclear	NR/unclear	Head-and-neck selected subgroup.	High
Pagnarith, 2010	Yes	N/A	Culture confirmed	N/A	N/A	Yes	NR/unclear	NR/unclear	Laboratory-based pediatric cohort.	Moderate
Pham, 2024	Unclear	N/A	Culture + molecular confirmation	N/A	N/A	Yes	NR/unclear	NR/unclear	Retrospective cohort.	Moderate
Pongrithsukda, 1988	Unclear	N/A	Culture (12/18) or historical IHA criteria	N/A	N/A	Yes	NR/unclear	NR/unclear	Historical mixed diagnostic criteria; greater misclassification risk.	High
Sam, 2006	Unclear	N/A	Culture confirmed	N/A	N/A	Yes	NR/unclear	NR/unclear	Retrospective pediatric cohort.	Moderate
The Lao Melioidosis Research Group, 2025	Yes	N/A	Culture confirmed	N/A	N/A	Yes	Adequate for in-hospital outcome	347/385 with known mortality	Mortality denominator explicitly restricted to patients with known outcome.	Low

Study	S1 Representative cohort	S2 Non-exposed cohort	S3 Melioidosis ascertainment	S4 Outcome absent at start	C1/C2 Comparability	O1 Outcome ascertainment	O2 Follow-up duration	O3 Follow-up completeness	Key note	Overall risk
Sitthikarnkha, 2025	Yes	N/A	Administrative ICD coding	N/A	N/A	Yes	Adequate for administrative outcome	Administrative completeness	Large national dataset but no uniform microbiological confirmation.	High
Smith, 2017	Unclear	N/A	Culture confirmed	N/A	N/A	Yes	NR/unclear	NR/unclear	Small pediatric subgroup.	Moderate
Suy, 2026	No/selected	N/A	Culture confirmed	N/A	N/A	Yes	NR/unclear	NR/unclear	Selected SST/head-and-neck cohort; outcome-specific denominators required.	High
Tauran, 2018	Unclear	N/A	Culture confirmed	N/A	N/A	Yes	NR/unclear	NR/unclear	Small multicentre case compilation.	Moderate
Turner, 2016	Yes	N/A	Culture confirmed	N/A	N/A	Yes	NR/unclear	NR/unclear	Retrospective pediatric cohort with clear clinical outcome reporting.	Moderate

Overall risk-of-bias rule: Low = no major concern and ≤1 unclear applicable item; Moderate = ≥2 unclear/minor concerns without a major concern; High = ≥1 major concern (e.g., selected/non-representative cohort, substantial diagnostic misclassification risk, or multiple serious limitations). N/A denotes structural non-applicability and is not a favorable judgment.
